# Maintenance of quantitative genetic variance in complex, multitrait phenotypes: the contribution of rare, large effect variants in 2 Drosophila species

**DOI:** 10.1093/genetics/iyac122

**Published:** 2022-08-12

**Authors:** Emma Hine, Daniel E Runcie, Scott L Allen, Yiguan Wang, Stephen F Chenoweth, Mark W Blows, Katrina McGuigan

**Affiliations:** School of Biological Sciences, The University of Queensland, Brisbane, QLD 4072, Australia; Department of Plant Sciences, University of California Davis, Davis, CA 95616, USA; School of Biological Sciences, The University of Queensland, Brisbane, QLD 4072, Australia; School of Biological Sciences, The University of Queensland, Brisbane, QLD 4072, Australia; Institute of Evolutionary Biology, University of Edinburgh, Edinburgh EH9 3FL, UK; School of Biological Sciences, The University of Queensland, Brisbane, QLD 4072, Australia; School of Biological Sciences, The University of Queensland, Brisbane, QLD 4072, Australia; School of Biological Sciences, The University of Queensland, Brisbane, QLD 4072, Australia

**Keywords:** mutation–selection balance, House of Cards, gene expression, genetic covariance, sparse factor analysis, standing genetic variance, *Drosophila serrata*, *Drosophila melanogaster*

## Abstract

The interaction of evolutionary processes to determine quantitative genetic variation has implications for contemporary and future phenotypic evolution, as well as for our ability to detect causal genetic variants. While theoretical studies have provided robust predictions to discriminate among competing models, empirical assessment of these has been limited. In particular, theory highlights the importance of pleiotropy in resolving observations of selection and mutation, but empirical investigations have typically been limited to few traits. Here, we applied high-dimensional Bayesian Sparse Factor Genetic modeling to gene expression datasets in 2 species, *Drosophila melanogaster* and *Drosophila serrata*, to explore the distributions of genetic variance across high-dimensional phenotypic space. Surprisingly, most of the heritable trait covariation was due to few lines (genotypes) with extreme [>3 interquartile ranges (IQR) from the median] values. Intriguingly, while genotypes extreme for a multivariate factor also tended to have a higher proportion of individual traits that were extreme, we also observed genotypes that were extreme for multivariate factors but not for any individual trait. We observed other consistent differences between heritable multivariate factors with outlier lines vs those factors without extreme values, including differences in gene functions. We use these observations to identify further data required to advance our understanding of the evolutionary dynamics and nature of standing genetic variation for quantitative traits.

## Introduction

The maintenance of quantitative genetic variance presents geneticists and evolutionary biologists with a formidable challenge. While models of the evolution of allele frequencies can be relatively simple, evolution of the genetic variance for phenotypic traits also depends on the effects of those alleles (Walsh and Lynch 2018). A substantial and complex body of theory has resulted in competing models, with no clear resolution of how quantitative variation evolves (Bürger 2000; Johnson and Barton 2005; Walsh and Lynch 2018). Despite the central importance of the nature of quantitative genetic variance both for predicting long-term phenotypic evolution (Arnold *et al.* 2008), and for optimizing approaches to identify causal loci (Eyre-Walker 2010; Simons *et al.* 2018), we have only limited empirical knowledge of the joint distribution of allele frequencies and their effects on traits of interest and on fitness (Johnson and Barton 2005; Walsh and Lynch 2018). Our purpose in this study is to revisit the basic observation of the distribution of genetic variance in complex quantitative phenotypes to help distinguish between the potential mechanisms underlying the maintenance of genetic variance. Our focus is on 2 key aspects of theoretical models: the relationship between allele frequency and effect size, and pleiotropy.

From a theoretical perspective, the resulting allele frequency spectrum is a key distinguishing feature between models in which selection actively maintains polymorphisms (balancing selection) and models in which selection eliminates variation (and mutation reintroduces it: mutation–selection balance, MSB). Balancing selection mechanisms are predicted to maintain relatively symmetrical allele frequencies at a locus, while under MSB models, genetic variation is determined by rare alleles, where the greater the fitness effect of a locus, the rarer the minor allele at that locus (Johnson and Barton 2005; Walsh and Lynch 2018). Genomic studies of adaptation have provided observations consistent with balancing selection models, such as fluctuation of allele frequencies with short-term environmental variation (e.g. Bergland *et al.* 2014), and the contribution to rapid adaptation of common, not rare, alleles (e.g. Kelly and Hughes 2019). On the other hand, large-scale genetic mapping studies in humans (Kemper *et al.* 2012; Zhao *et al.* 2016; Hernandez *et al.* 2019; Schoech *et al.* 2019) and other taxa (Josephs *et al.* 2015; Bloom *et al.* 2019) suggest a strong contribution to standing genetic variance of loci with rare alleles. These latter observations are consistent with MSB model predictions.

Different MSB models also make contrasting assumptions about the magnitude of effects of new mutations relative to segregating alleles: Gaussian models assume mutations arise frequently, but have small effects, while House-of-Cards (HoC) models assume rarer, larger effect mutations (Lande 1975; Turelli 1984, 1985; Walsh and Lynch 2018). GWAS of various human phenotypes suggest that genetic variance is due to additive effects of many loci of small effect (reviewed in Simons *et al.* 2018), consistent with the Gaussian model. In contrast, in a rare example of an explicit test of predictions of the alternative models, Hodgins-Davis *et al.* (2015) found strong support for the HoC model for gene expression traits across 3 taxa. Other analyses of gene expression data also highlight the strong contribution to heritable variation from rare alleles, indicating that the effect sizes of these rare alleles must be much larger than the effects of more common alleles (Kremling *et al.* 2018; Hernandez *et al.* 2019). Further evidence of mutational effects suggests that most new mutations contribute little phenotypic variation, with few (rare) mutations having large phenotypic effects (Mackay *et al.* 1992; Davies *et al.* 1999; Heilbron *et al.* 2014; McGuigan, Collet, McGraw *et al.* 2014), again more consistent with HoC models.

Notably, empirical observations of selection, mutation, and genetic variance are seemingly incompatible with any quantitative genetic theory on a trait-by-trait basis, leading to the incorporation of pleiotropy into theoretical models (Johnson and Barton 2005; Walsh and Blows 2009; Walsh and Lynch 2018). Although the empirical evidence for pleiotropy has been controversial (Paaby and Rockman 2013), advances in accessibility of genomic data, coupled with extensive phenotypic data, are revealing pleiotropic variants across diverse traits (Bulik-Sullivan *et al.* 2015; Chesmore *et al.* 2018; Geiler-Samerotte *et al.* 2020; Shikov *et al.* 2020). The empirical distribution of genetic variance in multiple traits presents a very different perspective on the maintenance of genetic variance than apparent when considering traits individually. While genetic variance in single traits appears essentially ubiquitous (Blows and Hoffmann 2005), most of the genetic variance in sets of multiple traits is typically restricted to a smaller subspace, defined by linear combinations of the measured traits (Kirkpatrick 2009; Walsh and Blows 2009; Blows and McGuigan 2015). This uneven empirical distribution of genetic variance across multivariate phenotypic space implies that the number of genetically independent traits (*n*) is much lower than the number of traits measured (*p*). Indeed, based on genetic load and genetic variance in fitness arguments, *n *>* *200 is predicted to be unlikely (Barton 1990; Johnson and Barton 2005). Hence, much of the genetic variation observed to be associated with an individual trait is expected to be shared with other traits.

Theoretical models differ in the assumptions about the correlation of pleiotropic effect sizes among traits, specifically, whether they are uncorrelated, or whether individuals that carry a pleiotropic allele that generates an extreme value for one trait will also be extreme for other traits (Turelli 1985; Barton 1990; Wingreen *et al.* 2003; Johnson and Barton 2005; Waxman and Peck 2006). While there is some evidence of stronger selection on mutations with highly pleiotropic effects (Denver *et al.* 2005; McGuigan, Collet, Allen *et al.* 2014), the distributions of allele frequency and of pleiotropic effects remain poorly characterized for any traits. Notably, theoretical models of MSB typically presume that mutations, while having negative effects on fitness, have unbiased effects on phenotypic traits (Johnson and Barton 2005). However, the emergence of extreme multivariate trait values from the pleiotropic effects on each individual trait is unknown. Here, we characterize the multivariate distribution of heritable phenotypes, aiming to interrogate the empirical relationship between variant frequency and effect size underpinning genetic covariances and to determine whether allelic effects on individual traits are predictive of the multivariate distribution of their effects across many traits.

To address these aims, we use high-dimensional Bayesian Sparse Factor Genetic (BSFG) modeling (Runcie and Mukherjee 2013) to interrogate the distribution of standing genetic variance in 2 unrelated datasets, one from *Drosophila serrata* (Allen *et al.* 2013; McGuigan, Collet, McGraw *et al.* 2014) and one from *Drosophila melanogaster* (Ayroles *et al.* 2009; Runcie and Mukherjee 2013). Separately for each species, we conduct analyses on datasets composed of 3,385 gene expression traits measured for each of 30 inbred lines, capturing standing genetic variance in the traits in the natural population from which flies were sampled. In both *D. serrata* and *D. melanogaster*, we confirm previous inferences of substantial genetic covariance of these gene expression traits (Ayroles *et al.* 2009; Runcie and Mukherjee 2013; Blows *et al.* 2015). We then investigate the distributions of this pervasive covariance using the heritable factor values estimated by the BSFG model. In both species, these analyses provide evidence that the standing genetic covariance of expression traits is largely determined by rare genetic variants of large effect.

## Methods

### *Drosophila serrata* dataset

A set of 30 highly inbred lines were derived by 15 generations of full-sib mating from a natural *D. serrata* population in Brisbane, Queensland, Australia, as detailed in Allen *et al.* (2013). Gene expression was measured for 2 biological replicates of males from each line (i.e. 60 samples in total), using a microarray approach (Allen *et al.* 2013). An earlier attempt at a high-dimensional analysis of these data was statistically limited to consider only one multivariate axis of trait variation (Blows *et al.* 2015). We now use Runcie and Mukherjee’s (2013) BSFG modeling to characterize genetic covariance more comprehensively in these data. We focus our analysis to a subset of 3,385 traits previously characterized for a different set of *D. serrata* lines (Hine *et al.* 2018).

### *Drosophila melanogaster* dataset

A set of 40 highly inbred lines were derived by 20 generations of full-sib mating from a natural *D. melanogaster* population in Raleigh, North Carolina, United States, and gene expression characterized as detailed in Ayroles *et al.* (2009). These lines are a subset of the *Drosophila melanogaster* Genetic Reference Panel, DGRP (Mackay *et al*. 2012). To facilitate comparability of the *D. serrata* and *D. melanogaster* datasets, we took a random subset of 30 of these *D. melanogaster* lines (sampling both biological replicates per line), and a random subset of 3,385 of the 10,096 genetically variable gene expression traits. We analyzed data for males only (as in *D. serrata*). The previous analysis of these data indicated strong patterns of genetic covariance among expression traits (Ayroles *et al.* 2009; Stone and Ayroles 2009). Runcie and Mukherjee (2013) previously subjected a much smaller subset (414) of these traits, implicated as being involved in competitive fitness, to BSFG modeling.

### Data distributions

As detailed in the original publications (Ayroles *et al.* 2009; Allen *et al.* 2013), expression was quantified using multiple probes per gene, and we follow the original authors in analyzing the median expression of these probes (i.e. one estimate per gene). The *D. melanogaster* was log_2_ transformed (Ayroles *et al.* 2009), while the *D. serrata* median expression was on a log_10_ scale as in McGuigan, Collet, McGraw *et al.* (2014). The BSFG model (Runcie and Mukherjee 2013) (detailed below) was fit to z-scores (i.e. the trait mean was subtracted from the observation, and these centered values were divided by the trait-specific standard deviation). Before fitting the model, we investigate the distribution of these data and consider them on both this z-score (SD) scale and on the IQR scale (i.e. the trait median was subtracted, and these centered observations divided by the trait-specific IQR). On each scale, we consider the distribution of both phenotypic and genetic (line-mean) trait values. We compared the observed data to simulated data, generated by independently sampling 3,385 sets of 60 values from a normal distribution using the rnorm function in R (R Core Team 2021). The simulated data were centered and scaled with respect to their trait-specific means or medians and SD or IQR.

**Fig. 1.**
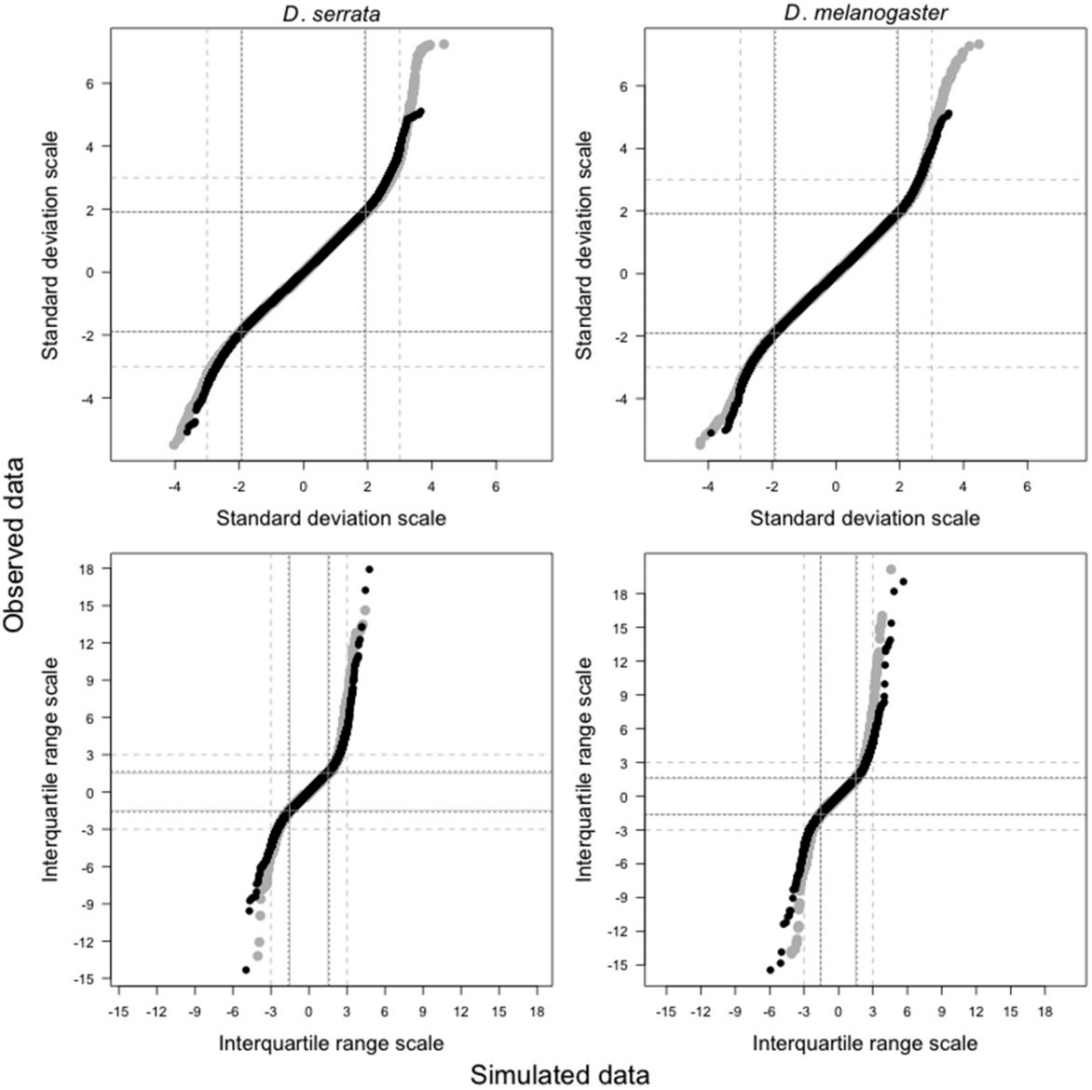
Quantile–quantile plots of observed data vs simulated normally distributed data. For each dataset (*D. serrata*, left column and *D. melanogaster*, right column) each individual gene expression trait was centered and scaled to its own mean and standard deviation (SD scale, top row) or median and IQR (scale, bottom row). All 201,300 phenotypic (grey) or 101,550 genetic (black) values were then pooled and sorted. On both scales, the distributions of the middle 95% of values were in close agreement between the observed data (*y*-axis) and simulated normal data (*x*-axis). Horizontal (vertical) lines demarcate 2.5–97.5% of the observed (simulated) data; these quantiles were indistinguishable between phenotypic (solid grey lines) and genotypic (dotted black lines) values. Dashed grey lines demarcate ±3 units on either scale; on the SD scale this corresponds to a common threshold for identifying outliers, and on the IQR scale is the threshold used in the current study to identify extreme values.

For each dataset and scale, we pooled the 203,100 scaled values (or the 101,550 scaled line means) across traits, then plotted the sorted pooled observed values against the sorted pooled values from the simulated data (Fig. 1). The observed data of both species were largely consistent with a normal distribution, with 94.2–95.4% of the observed values falling within the middle 95% of the simulated values (Fig. 1). However, the ∼5% of trait values in the tails of the distributions were of larger absolute magnitude than expected for normally distributed data (Fig. 1). These characteristics of our data are consistent with general trends for gene expression data: Liu *et al.* (2019) reported that while most of 100 GEO or NCBI gene expression datasets from 20 species were normally distributed, many followed a t-distribution, which is similar to the normal distribution but with heavier tails (i.e. extreme values occur more frequently). We further consider the distribution of the data below, after outlining the analyses.

### Statistical analysis

We employed the BSFG model described by Runcie and Mukherjee (2013) to partition the variance among and within lines. Briefly, this approach aims to overcome the twin challenges of analyzing phenotypic data when the number of traits is large, and the partitioning of that phenotypic variation to genetic and environmental sources. This analytical tractability is achieved by assuming that the genetic covariance matrix, **G**, is modular and of low rank (i.e. sparse). This assumption is consistent with published estimates of **G** from a range of taxa and traits that suggest both modularity (Wagner and Zhang 2011) and reduced rank (Blows and McGuigan 2015) are common characteristics of **G**. Gene expression traits, because they are functionally connected through regulatory networks (Davidson and Levin 2005), may be expected to be particularly characterized by modularity and low dimensionality.

The BSFG analysis is founded in the classical linear mixed model that partitions observed phenotypic variation to genetic and nongenetic sources. Here, the linear mixed model, fit separately to the *D. serrata* and to the *D. melanogaster* data, is:

(1) Y=XB+ZU+E,

where **Y** is the 60 × 3,385 matrix of observations for the 60 (30 lines by 2 replicates) measures of the 3,385 gene expression traits; **X** is the 60 × 1 incidence matrix relating observations to the fixed effect of trait means where **B** is the 1 × 3,385 matrix of trait means; **Z** is the 60 × 30 incidence matrix relating the 2 replicates per line to their respective line; **U** is the 30 × 3,385 matrix such that *u_it_* is the mean effect of line *i* on trait *t*; and **E** is the 60 × 3,385 matrix of deviations of the replicates from their line mean, such that *e_ijt_* is the effect of replicate *j* of line *i* on trait *t*.

The random effects of genotype (line) and replicate (reflecting microenvironment and measurement error), **U** and **E**, are assumed to follow multivariate normal distributions:

(2) $$u_{i.}∼{\text{MVN}}_{3385}\left(0,G\right)$$

(3) $$e_{ij.}∼{\text{MVN}}_{3385}\left(0,R\right),$$

where the genetic (**G**) and replicate (**R**) variance–covariance matrices sum to give **P**, the phenotypic variance–covariance matrix:

(4) P=G+R.

In the BSFG model, common and specific components of phenotypic (co)variance are estimated by modeling **P** as:

(5) $$P=\Lambda \Lambda^{T}+\Psi ,$$

where **Λ** is the 3,385 × *k* matrix whose columns form the *k* predicted latent factors underlying the phenotypic variance and **Ψ** is the diagonal matrix containing the 3,385 trait-specific variances.

In the BSFG algorithm, the value of *k* is free to vary from one iteration of the Gibbs sampler to the next, unless otherwise specified. For computational efficiency, we constrain the model to estimate at most 59 factors; this number corresponds to the maximum number of nonzero eigenvalues in an orthogonal decomposition of a covariance matrix of 60 observations per trait. BSFG analysis is not well suited to estimating the true number of independent latent traits in the data (i.e. the true value of *k*), and is instead primed to provide robust estimates of latent factors with the greatest influence on the total phenotypic variation (Runcie and Mukherjee 2013). Underlying this treatment of *k* is the “infinite factor model” of Bhattacharya and Dunson (2011), which allows *k* to be infinite but forces the variances of loadings of higher-order factors to stochastically decrease toward zero. A related approach as proposed by Knowles and Ghahramani (2011) is to allow *k* to be infinite but enforce that the proportion of elements of each vector of trait loadings stochastically increases. Both approaches allow truncation of higher-order factors for computational convenience without compromising model fit and permit simpler computational algorithms for model inference.

A second point of difference between BSFG and other mixed-effect factor models, which increases the biological interpretability of BSFG estimates, is that the factors estimated in the BSFG are not constrained to be orthogonal. Instead, each trait loading is modeled directly under the sparsity prior (Supplementary Table 1), allowing interpretation of the biological modules underlying the estimated factors (Runcie and Mukherjee 2013).

The common (i.e. **ΛΛ**^T^) and specific (i.e. **Ψ**) components of **P** can each be partitioned into genetic and nongenetic components:

(6) $$G={\Lambda \Sigma }_{{\Lambda h}^{2}}\Lambda^{T}+\Sigma_{{\Psi h}^{2}}\Psi$$

(7) $$R={\Lambda (I_{k}-\Sigma }_{{\Lambda h}^{2}})\Lambda^{T}+(I_{p}-\Sigma_{{\Psi h}^{2}})\Psi ,$$

where $\Sigma_{{\Lambda h}^{2}}$ is the *k × k* diagonal matrix of latent factor heritabilities, $\Sigma_{{\Psi h}^{2}}$ is the 3,385 × 3,385 diagonal matrix containing heritabilities of the specific variances, and **I**_*k*_ and **I**_*p*_ are identity matrices of size *k* and *p*, respectively. A summary of the outlined parameters can be found in Table 1, and the prior distributions they were modeled under in Supplementary Table 1. As detailed above, the BSFG analysis was implemented on variance-standardized data, which allowed us to interpret each squared trait loading, λ^2^_*ij*_, as approximately the proportion of phenotypic variance in trait *i* that can be explained by factor *j*.

**Table 1.** Description of datasets and summary of estimated and derived parameters.

Category	Term and description/relation to model
Types of data	*Observed data:* The *n *=* *60 observations of the *p *=* *3,385 expression traits, appearing in the model as the *n* × *p* matrix **Y**. There are 2 observed datasets, one for each species.
	*Randomized data:* The 100 permutations of the observed data. For each randomized dataset, the 30 pairs of observations (2 replicates per line) per trait were shuffled independently for each trait, randomly reassigning the replicate measurements per line. This retains the distributions of phenotypic and genotypic values for each trait, whereas covariance between traits is the result of sampling error and not biologically meaningful.
	*Simulated data:* We sampled 3,385 sets of 60 values from a normal distribution. These data are presented in Fig. 1 only.
	*Adjusted data:* The remaining trait value, **Y**_specific_, after accounting for the predicted contribution of the latent traits (**FΛ**^T^) on the observed gene expression phenotypes (**Y**)**:Y**_specific_ = **Y—FΛ**^T^.
Estimated parameters	*Latent factors:* The columns of the *p* x *k* matrix **Λ**.
	*Latent trait values:* The *n* × *k* matrix **F** contains the predicted latent trait values for each sample such that the *n* × *p* data matrix **Y** can be expressed in terms of common and specific contributions to each phenotype: **Y**=**Y**_common_ + **Y**_specific_, where **Y**_common_=**FΛ**^T^.
	*Trait loadings:* The individual elements of **Λ** such that *λ_ij_* represents the effect of the *j*th factor on the *i*th trait.
	*Factor heritability:* The heritability of latent trait *j* forms the *j*th diagonal element of the *k* × *k* diagonal matrix $\Sigma_{{\Lambda h}^{2}}$.
	*Specific variance:* Represented in the model by the parameter **Ψ**, the diagonal matrix with *p* diagonal elements corresponding to the specific variances of the *p* traits. In this study, the focus is not on **Ψ** per se. Instead, we examine the adjusted data to determine to what extent the latent factors account for the extreme values in the observed gene expression phenotypes.
	*Specific heritability:* Represented in the model by the parameter $\Sigma_{{\Psi h}^{2}}$, the diagonal matrix containing the heritabilities corresponding to the *p* specific variances. Again, this parameter was not a focus of our investigations; we instead compare the frequency of outlier lines between the observed and adjusted data.
Significance testing	*LFSR:* Analogous to a *P*-value, the probability of assigning the incorrect sign to an estimate. For trait loadings, this was the proportion of posterior samples of the trait loading that were equal to zero, or on the other side of zero from the median of the posterior samples. For factor heritabilities, which were constrained to be non-negative, this was calculated as the number of posterior samples equal to zero.
	*Average error rate (s):* Analogous to Storey’s *q*-value (Storey 2003; Stephens 2017). The relevant set of LFSRs (e.g. for the *p* trait loadings within a factor or the *k* factor heritabilities) is sorted in ascending order in a vector ***x***, where the *j*th unsorted LFSR corresponds to $x_{i}$. The average error rate for the *j*th estimate in the set is then $s_{j}=\frac{1}{i}\sum_{1}^{i}x_{i}$.
	*Number of significant trait loadings* (*n*_tt_): For a given factor, the number of trait loadings that are significant at *s* < 0.005 (2-tailed test as loadings can be positive or negative).
	*Statistically supported factors:* Columns of **Λ** with *n*_tt_ ≥ 2.
	*Heritable factor:* A factor with heritability significant at *s* < 0.01 (1-tailed test as variances cannot be negative).
Data interrogation	*Extreme values:* Values more than 3 trait-specific IQRs from the trait-specific median.
	*Outlier line:* For a given observed or latent trait, a line that meets 3 criteria: (1) the mean of its 2 replicate measurements exceeds 3 IQR from the median of the 30 lines; (2) both replicates of the line exceed 3 IQR from the median of the 60 observations per trait; and (3) both replicates deviate in the same direction from the median.
	*Number of significantly loading traits with outlier lines* (*n*_ot_): For a given factor, the number of traits that load significantly onto the factor and are associated with one or more outlier lines.

Prior distributions corresponding to the estimated parameters can be found in Supplementary Table 1.

### Convergence diagnostics

To determine whether the model had converged, we considered autocorrelation across posterior samples for each estimated parameter. Approximately 1.5% of the trait loading estimates were associated with posterior autocorrelations exceeding 0.1. However, we were satisfied that the model had achieved a sufficient degree of convergence to proceed with interpretation, as posterior means for trait loadings and latent trait values were largely unchanged between sets of posterior samples taken after a burn-in period of 1,000,000 or 2,000,000 samples, as well as a previous implementation of the model in MATLAB (not shown).

### Significance testing

To determine statistical significance of the latent factors and their heritabilities, we used the local false sign rate (LFSR) approach with an average error rate of 1%, as described in Hine *et al.* (2018; Table 1). We tested the significance of the factors via the trait loadings (Table 1) and considered a factor to be statistically supported when at least 2 trait loadings were statistically significant. To test whether the heritability estimate of a factor was significantly greater than zero, we again used an LFSR test, and error rate correction (Table 1), but imposed additional requirements due to the potential for spurious associations in these data (randomization analysis, detailed below).

### Identification of outlier lines

Other studies investigating the relationship between allelic effect size and frequency have defined effect size quantitatively (e.g. as the rank: Kremling *et al.* 2018), or qualitatively by defining outlier genetic variants based on a threshold z-score (reviewed in Table 1 of Richter *et al.* 2019). As outlined above, BSFG analyses were conducted on z-scores. However, we observed that when multiple visibly extreme values were present for a given trait, the inflation of variance due to the most extreme value sometimes resulted in z-scores of less extreme (but still visibly outlying) values falling within ±3 SD, a common threshold for identifying outliers. To ensure that we were capturing all extreme values in our identification of outliers, we instead quantified effect size in units of IQR from the trait- (or factor-) specific median (Fig. 1) and interrogated the distributions of individual gene expression traits and latent traits on this IQR scale. As noted above, the majority of the observed trait values closely match normally distributed data on both the z-score and IQR scales, but the deviation of the tails is more pronounced on the IQR scale (Fig. 1), facilitating identification of extreme observations.

For individual gene expression trait measurements (Supplementary Table 2), and for latent trait values from the BSFG model (Supplementary Figs. 1 and 2), we characterized a line as an outlier if each of 3 criteria were satisfied (Table 1). First, the mean of the 2 replicate values per line was greater than 3 IQR from the median of the 30 line means for that trait. Second, both replicate values per line were greater than 3 IQR from the median of the 60 values for that trait. Third, line replicate values were extreme deviates in a consistent direction (i.e. both replicates of the line fell on the same side of the median). The second and third criteria ensured that we were uncovering extreme genetic variants, not extreme values generated by spurious microenvironmental or technical effects on some individuals.

### Determination of spurious patterns through data randomizations

For datasets such as ours, where the number of variables (3,385) is far greater than the number of objects measured per variable (60 samples), random correlations among variables are expected to be common (e.g. Johnstone 2001). To help assess the biological relevance of the estimated factors, we implemented the BSFG model on randomized versions of the data from each species. Specifically, we shuffled the observations independently for each trait, randomly reassigning the pair of replicate measurements per line, and retained 100 such randomized datasets per species. This randomization approach retains the exact distributions and heritability of individual traits while generating a null genetic covariance among traits. That is, the randomization simulates data in which the true **Λ** in equation (6) is zero, allowing us to characterize the effect of sampling error on the estimation of **Λ**. Here, we focus on the effect of sampling error on genetic, rather than phenotypic, covariance. This is a conservative approach as, by retaining replicate pairs within each line, we inflate the potential for heritable covariance above that occurring if replicates were randomly assigned to a line.

**Fig. 2.**
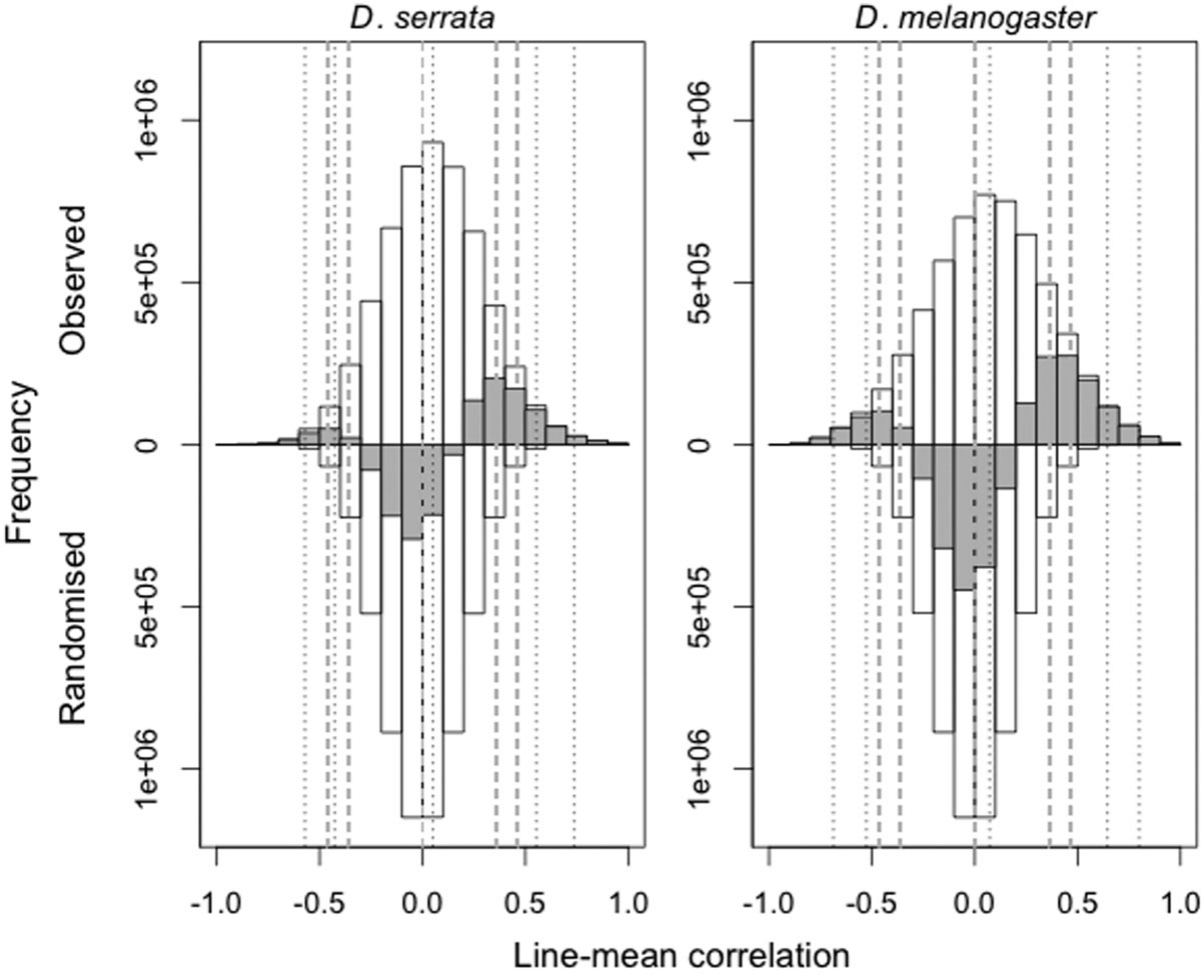
Distributions of the 5,727,420 pairwise genetic (line-mean) correlations of gene expression traits in observed and randomized data for *D. serrata* (left) and *D. melanogaster* (right). Genetic correlations for the observed (randomized) data correspond to white bars above (below) *y* = 0. The frequencies shown for the randomized datasets are averaged across the 100 datasets. Grey bars show the difference in frequency between the observed and randomized data [i.e. above (below) *y* = 0 indicate inflation (deflation) in the observed relative to the randomized]. Vertical lines indicate the quantiles 0.005, 0.025, 0.500, 0.975, and 0.995 for the observed (dotted black lines) and randomized (dashed grey lines) datasets.

As anticipated, sampling error generated spurious genetic correlations among traits (Fig. 2; Supplementary Table 3), resulting in factors that were significantly, but spuriously, heritable in the randomized datasets. These patterns reflect the substantial opportunity for sampling error within these data (with ∼112 times more traits than independent genetic observations per trait), but also the inherent biology in the data. Many expression traits had at least one outlier line (Supplementary Table 2). As a result, random shuffling of traits could result relatively frequently in a random “line” having extreme values of multiple expression traits, causing substantial pairwise line-mean correlations, and significantly heritable factors.

Notably, there were 10 (*D. serrata*) or 20 (*D. melanogaster*) times as many large pairwise correlations (i.e. |*r*| > 0.5) in the observed data than the randomized datasets (Fig. 2, Supplementary Table 3). The randomized and observed data also differed in the median pairwise genetic correlation, which was zero for the randomized data, but positive for the observed data; this was reflected in the greater deviation of observed data above random expectation for positive correlations (Fig. 2; Supplementary Table 3). Because the individual trait distributions are identical between the observed and randomized datasets, the greater frequency of higher magnitude correlations in the observed data demonstrates that a substantial amount of covariance in the observed data reflects biological signal above that generated by random cosampling of genotypes. We therefore established criteria based on associations between data parameters to allow us to identify factors in the observed data that were capturing more than spurious associations among traits.

In each species, we observed a linear relationship between the total number of significant trait loadings (*n*_tt_; Table 1) and how many of those significantly loading traits had at least one outlier line (*n*_ot_; Table 1; Supplementary Fig. 3). This pattern is consistent with the expectation that the biological signal in the data (outlier lines for individual traits) could result in the estimation of heritable factors that merely reflect chance covariance arising from cosampling of extreme values. We used *n*_tt_ and its observed relationship with *n*_ot_ to assess whether observed heritable factors were likely to reflect biological signal. First, we considered any observed heritable factor to reflect biological signal if its *n*_tt_ exceeded the maximum *n*_tt_ across species-specific randomized data heritable factors. Most (29 of 35) *D. serrata* heritable factors but only 4 of 42 *D. melanogaster* heritable factors were retained for further analysis based on this criterion (Supplementary Tables 4 and 5). Second, for observed heritable factors that did not meet the first criterion, we considered the relative number of *n*_ot_ given *n*_tt_. Specifically, we compared each observed heritable factor to the subset of randomized data heritable factors with the same *n*_tt_. If the observed *n*_ot_ fell outside the 95% range for randomized heritable factors with the same *n*_tt_, we retained it for further investigation. A further 3 *D. serrata* and 18 *D. melanogaster* heritable factors were retained based on this second criterion (Supplementary Fig. 3). The remaining heritable factors were not considered further, as their trait compositions were not distinguishable from those of randomized data heritable factors.

### Imposed directionality of latent traits

Like an eigenvector, the direction of each factor (column of **Λ**) is arbitrary. To aid interpretation of the latent traits, we imposed directionality on each factor by multiplying its trait loadings and latent trait values by the sign of its mean trait loading (i.e. by +1 or −1, depending on whether the average loading was positive or negative). This allowed us to investigate whether extreme variants were more likely to be associated with an overall increase or decrease in gene expression across the variance-standardized traits significantly affected by the latent factor.

### Investigating potential causes of covariation in expression

Genetic covariance in gene expression, as with other traits, can arise due to pleiotropy or linkage. Pleiotropic loci affecting expression of large numbers of genes (e.g. Wang *et al.* 2010; Lukowski *et al.* 2017) have been identified, and as such it is plausible that heritable factors reflect among-line allelic divergence at pleiotropic loci. However, the experimental data analyzed here cannot directly determine the genetic variant(s) responsible for the coexpression revealed by the BSFG, and thus whether pleiotropy is responsible. We can further address the alternative hypothesis that genes coassociated with a heritable factor are colocalized in the genome, reflecting physical linkage disequilibrium.

Allen *et al.* (2013) mapped 95% of all expressed sequence tags (ESTs) on the *D. serrata* microarray analyzed here to *D. melanogaster* chromosomes. Subsequent publication of the *D. serrata* genome assembly (available on NCBI: BioProject: PRJNA355616; Allen *et al.* 2017) was consistent with the previous inference of strong gene location conservation between *D. serrata* and *D. melanogaster* (Stocker *et al.* 2012). A more recent scaffolding using DovetailTM Hi-C technology (Dovetail Genomics) greatly improved the contiguity of the assembly from an N50 of just under 1 Mb (Allen *et al.* 2017) to an N50 of 30.3 Mb (Allen S, personal communication). Chromosome locations of the constructed scaffolds were determined based on 78 physical and linkage markers with known chromosome location (as identified by Stocker *et al.* 2012).

We queried the sequences of the 3,385 ESTs against the scaffolded *D. serrata* reference using the default settings in BLASTN (version 2.2.27+; Altschul *et al.* 1990). This resulted in 94% of the ESTs mapping to the chromosomes X, 2L, 2R, 3L, and 3R, which were, respectively, associated with 446, 545, 683, 688, and 817 ESTs. ESTs that did not successfully align with the reference genome were categorized as “other,” and are likely to have been derived from genes on chromosomes 4 or Y. We assumed the frequencies with which ESTS mapped to chromosomes were representative of an underlying multinomial distribution and used chi-square tests to determine whether subsets of ESTs corresponding to each heritable factor were distributed nonrandomly across chromosomes.

To investigate the potential role of linkage disequilibrium in the identification of *D. melanogaster* heritable factors, we focused specifically on the inversions that have been identified for these lines (Huang *et al.* 2014). Inversion genotypes (downloaded from: http://dgrp2.gnets.ncsu.edu/data.html) were available for 29 of the 30 lines analyzed here. Across these 29 lines, 5 known inversions were segregating, present as 1 or 2 copies across 11 lines (including one line carrying 2 inversions). Two of the 5 inversions, *In(2L)t* and *In(3R)Mo*, are associated with variation in the expression levels of hundreds of genes (Lavington and Kern 2017). Here, we were particularly interested in whether large changes in gene expression (i.e. heritable factors with outliers) could be attributed to the presence of any of these inversions.

### Investigating differentiation in genetic roles of different classes of heritable factor

We conducted functional enrichment analyses on the subsets of genes represented by heritable factors. For *D. melanogaster*, we used FlyBase Gene IDs corresponding to the Affymetrix probe set IDs as listed on the Gene Expression Omnibus (accession number GPL1322). Of the 3,385 probe sets analyzed, 3,119 were associated with a single FlyBase Gene ID (110 had none, while the 156 probe sets associated with multiple FlyBase Gene IDs were also omitted). For *D. serrata*, we used FlyBase gene IDs corresponding to orthologs in *D. melanogaster* of the *D. serrata* transcript-level coding sequence, matched to *D. serrata* ESTs using BLASTN (default settings, version 2.2.27+; Altschul *et al.* 1990). Orthologs were assigned using OrthoDB (Kriventseva *et al.* 2019). Transcript-level coding sequence was extracted from the annotation GFF file at NCBI *D. serrata* Annotation Release 100 (https://www.ncbi.nlm.nih.gov/genome/annotation_euk/Drosophila_serrata/100/) using GffRead (Pertea and Pertea 2020). At least one ortholog was identified for 2,486 of the 3,385 *D. serrata* ESTs, including 5 ESTs with multiple orthologs and 90 orthologs that were represented by between 2 and 4 ESTs, resulting in 2,154 unique *D. melanogaster* orthologs. Enrichment analyses were conducted using the *gost* function in the *gprofiler2* R package (Raudvere *et al.* 2019) using the false discovery rate method of multiple testing correction with a threshold of 0.05. Queries were made against annotated genes from custom backgrounds corresponding to the 3,119 (*D. melanogaster*) or 2,154 (*D. serrata*) FlyBase Gene IDs. Limiting the background to only the genes that could have been in the gene set of interest reduces the probability of detecting significant enrichment where there is none (Timmons *et al.* 2015). We implemented semantic similarity analysis on any identified enriched terms for each heritable factor using the R package GOSemSim (Yu *et al.* 2010).

## Results

**Fig. 3.**
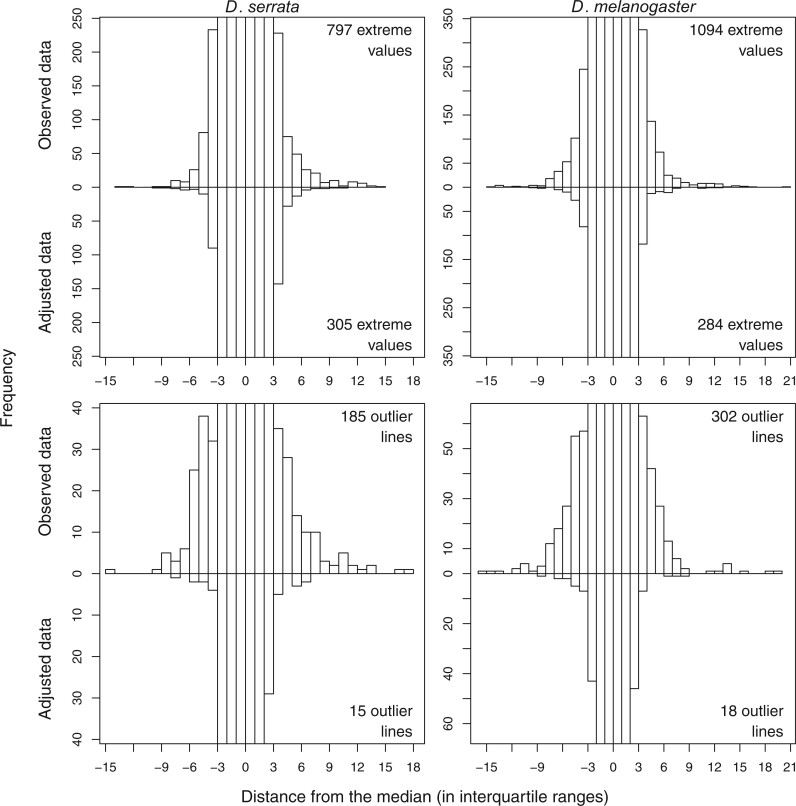
Observed trait distributions before and after adjusting for the predicted contribution of latent factors in *D. serrata* (left panels) and *D. melanogaster* (right panels). Top row: Distribution of the 203,100 (60 observations × 3,385 traits) IQR-scaled values in the observed data (above *y* = 0) and the adjusted data (defined in Table 1; below *y* = 0). Bottom row: Distribution of the 30 IQR-scaled line means for the 132 traits in *D. serrata* (3,960 observations) and the 228 traits in *D. melanogaster* (6,840 observations) that were associated with at least one outlier line (defined in Table 1; Supplementary Table 2). Counts of extreme values (top row) and outlier lines (bottom row) in the observed and adjusted data are shown within each panel. To facilitate the comparison of the tails of the distributions, the *y*-axis range for each panel has been truncated at the maximum count for values more than ±3 IQR from the median in each panel (i.e. values between −3 and 3 on the *x*-axis extend beyond the shown limit on the *y*-axis).

The BSFG analysis returned evidence consistent with previous analyses of these data, namely that a substantial portion of the variation in expression was shared among traits, not trait specific. In *D. serrata*, all 59 (i.e. the maximum specified) factors were statistically supported, collectively explaining 59% of the total phenotypic variance in the 3,385 expression traits, while in *D. melanogaster* 39% of the phenotypic variance was explained by 47 statistically supported factors. We assessed the effect of these latent traits on the distribution of gene expression phenotypes and, in particular, whether the observed extreme gene expression values (Fig. 1) were accounted for by shared variation (i.e. these factors) or instead reflected trait-specific effects. To do this, we adjusted the observed data by subtracting the predicted contribution of the latent traits to the observed traits (Table 1), and centered and scaled these adjusted values by their trait-specific medians and IQR. Comparing the IQR-scaled outlier-line traits before and after fitting the BSFG model (Fig. 3, top row), we found that there were approximately 62% (74%) fewer extreme values in the adjusted data in *D. serrata* (*D. melanogaster*). That is, latent factors contributed more than trait-specific effects did to extreme values in the data.

In *D. serrata* and *D. melanogaster*, respectively, 32 and 22 of the latent factors were significantly heritable and met the additional criteria from the randomization analysis (Supplementary Fig. 3). In *D. serrata*, heritability estimates for these heritable factors ranged from 0.33 to 0.99 (Supplementary Fig. 1), with individual heritable factors significantly influencing between 4 and 483 traits (Supplementary Table 4). In *D. melanogaster*, factor heritability similarly ranged from 0.30 to 0.99 (Supplementary Fig. 2) with between 5 and 549 traits significantly influenced by a heritable latent factor (Supplementary Table 5). We assessed the effect of the heritable latent traits on the distribution of genotypic values for the gene expression traits. Comparing the IQR-scaled genotypic values of outlier-line traits before and after fitting the BSFG model (Fig. 3, bottom row), we found that a 92% (*D. serrat*a) or 94% (*D. melanogaster*) reduction in extreme genotypic values after model fit. This suggests that the majority of extreme genotypic values may arise via processes affecting multiple traits.

**Fig. 4.**
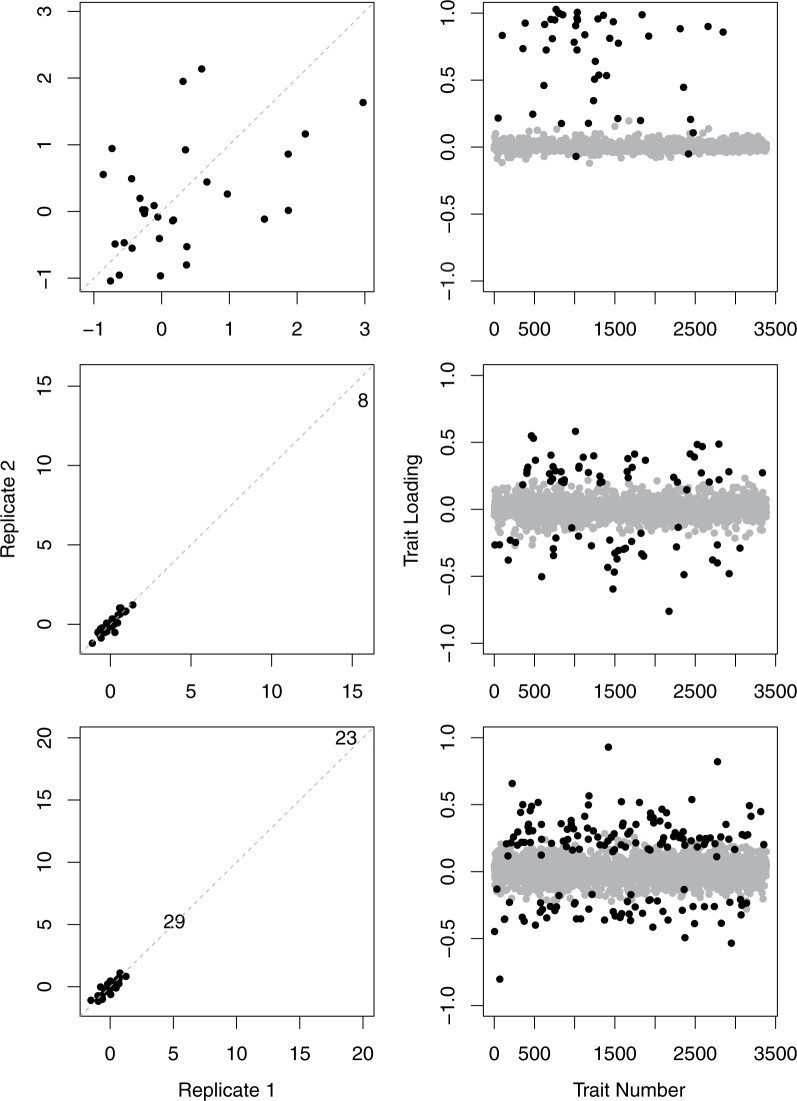
Example heritable factors from the *D. serrata* dataset. Left hand column shows the distribution of estimated latent trait values of the 30 lines (points) on the IQR scale for each of the 2 replicates per line. The dashed line indicates a 1:1 relationship between replicate latent trait value IQR deviations. The corresponding trait loadings for these heritable factors are also illustrated (right column): black (grey) circles depict significant (nonsignificant) trait loadings. Traits are ordered by numerical identifiers that were arbitrarily assigned before analyses. Heritable factor 26 (top row) had no outlier lines, HF 19 (middle) had one outlier line (8) and HF 6 (bottom) had 2 outlier lines (23 and 29). Further details on these factors in Supplementary Fig. 1 and Supplementary Table 4.

Inspection of the distributions of genotypic values for each of the 32 *D. serrata* and 22 *D. melanogaster* latent factors for which heritability was statistically supported revealed patterns that suggested only some of the heritable factors may contribute to the extreme genotypic values. For 7 (22%) and 11 (50%) of the heritable factors in *D. serrata* and *D. melanogaster*, respectively, there were no outlier lines (Supplementary Figs. 1 and 2; e.g. Fig. 4, top left). For the other 25 (78%) and 11 (50%) heritable factors in *D. serrata* and *D. melanogaster*, respectively, there was at least one outlier line (Supplementary Figs. 1 and 2; Fig. 4, center and bottom left). Every *D. serrata* line was an outlier for at least one heritable factor, while 8 lines were extreme for 2 and 1 line was extreme for 3 heritable factors, resulting in 40 outlier line values (4.2%) across the 960 (30 lines × 32 heritable factors) latent trait values (Supplementary Fig. 1). Most (14) of these 25 heritable factors with extreme values had only one outlier line (Supplementary Fig. 1; e.g. Fig. 4, center left), but some had 2 or 3 (7 and 4 heritable factors, respectively; Supplementary Fig. 1; e.g. Fig. 4, bottom left). In *D. melanogaster*, there were 11 extreme values (1.7%) across the 660 (30 lines × 22 heritable factors) latent trait values, with at most a single extreme value for any given line or heritable factor (Supplementary Fig. 2).

The joint observations that extreme trait observations reflected trait covariance (Fig. 2) and that a subset of heritable latent factors was associated with outlier values (Supplementary Figs. 1 and 2) suggests that it is the latent factors with outlier genotypic values that result in outlier genotypic values in individual traits. We investigated this taking 2 approaches. First, we confirmed that heritable factors with outliers were disproportionately associated with outlier line values for individual traits. Of the 3,385 traits measured, 3.9% (*D. serrata*) or 6.7% (*D. melanogaster*) had at least one outlier line (Supplementary Table 2), with some lines deviating up to 18 (*D. serrata*) or 19 (*D. melanogaster*) IQR from the median (Fig. 1). In *D. serrata*, 132 individual expression traits had outlier line(s); 127 of these traits were also associated with heritable factors with outlier line(s) (Supplementary Table 2). Similarly for *D. melanogaster*, traits with outliers were disproportionately associated with heritable factors with outliers, although the relationship was not as definitive: for the outlier traits associated with any heritable factor, 64.5% of outliers were influenced by heritable factors with an outlier line, while 20.1% were influenced by heritable factors without an outlier line (Supplementary Table 2; the remaining 15.4% were influenced by both types of heritable factor).

**Fig. 5.**
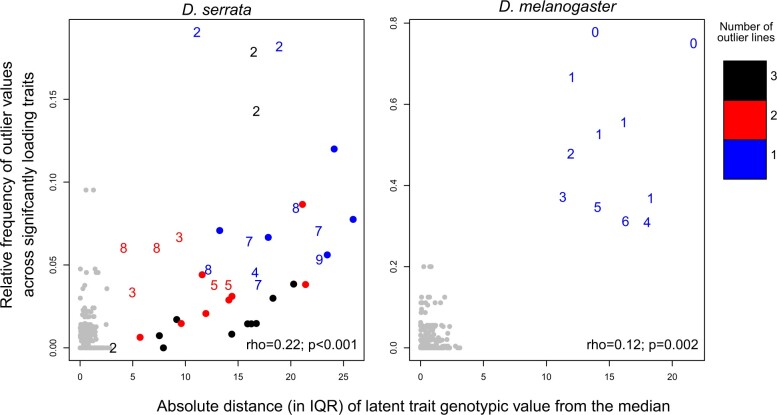
Prediction of observed trait outliers from latent trait values. For traits significantly influenced by a specific factor, for each line we calculated the relative frequency of outlier values (*y*-axis; note that scales differ between panels). This value was plotted against the magnitude of the latent trait value per line (*x*-axis), revealing a significant correlation (Spearman’s correlation statistics in bottom right of each panel). Plot symbols (numbers) indicate the number of significant trait loadings for the factor, in steps of 10, from “0” indicating a factor with <10 loadings through to a dot for >100 loadings (Supplementary Tables 4 and 5). Plot colors indicate the number of outlier lines for that factor (see figure for key). For example, a red “3” indicates a factor with 2 extreme line-mean values (red) and between 30 and 39 significant trait loadings (“3”). All lines with nonextreme latent trait values (<3 IQR from the median) are shown in grey, including all 30 lines for those factors with no outlier lines, and the 27–29 nonoutlier lines for factors with at least one outlier line (Supplementary Figs. 1 and 2).

Second, we investigated whether the frequency of outlier trait values for individual lines could be predicted by the magnitude of the latent trait value. We observed a statistically significant but imperfect association (Fig. 5). Individual lines that were not outliers on a given heritable factor (grey points <3 IQR on the *x*-axis in Fig. 5) rarely had outlier values of any associated individual trait (i.e. have a value of 0 on the *y*-axis in Fig. 5), while lines that were outliers for a heritable factor (colored points >3 IQR on the *x*-axis in Fig. 5) tended to also be outliers for a higher proportion of the traits influenced by that factor (higher values on *y*-axis in Fig. 5).

While there was a strong association between outlier lines for latent heritable factors and individual traits, there were also exceptions. First, not all traits with outlier lines were accounted for by factors (Supplementary Table 2), indicating trait-specific genetic effects of large magnitude. Second, some traits with outlier lines were associated with both types of heritable factor (i.e. with and without outlier lines; Supplementary Table 2); this is reflected in some lines that were not outliers for a given factor being outliers for up to 10% (*D. serrata*) or 20% (*D. melanogaster*) of the associated individual traits (grey points with values > 0 on the *y*-axis in Fig. 5). Third, most *D. serrata* line(s) that were outliers for a heritable factor were outliers for only a small proportion (<10%) of the associated individual traits (Fig. 5), suggesting that latent factors do not consistently cause outlier values of all influenced traits. To summarize, evidence from both species was consistent with some latent factors with outlier line values accounting for heritable extreme values of individual expression traits, although extreme values for the line on the heritable factor did not inevitably result in all coinfluenced traits having extreme values.

**Fig. 6.**
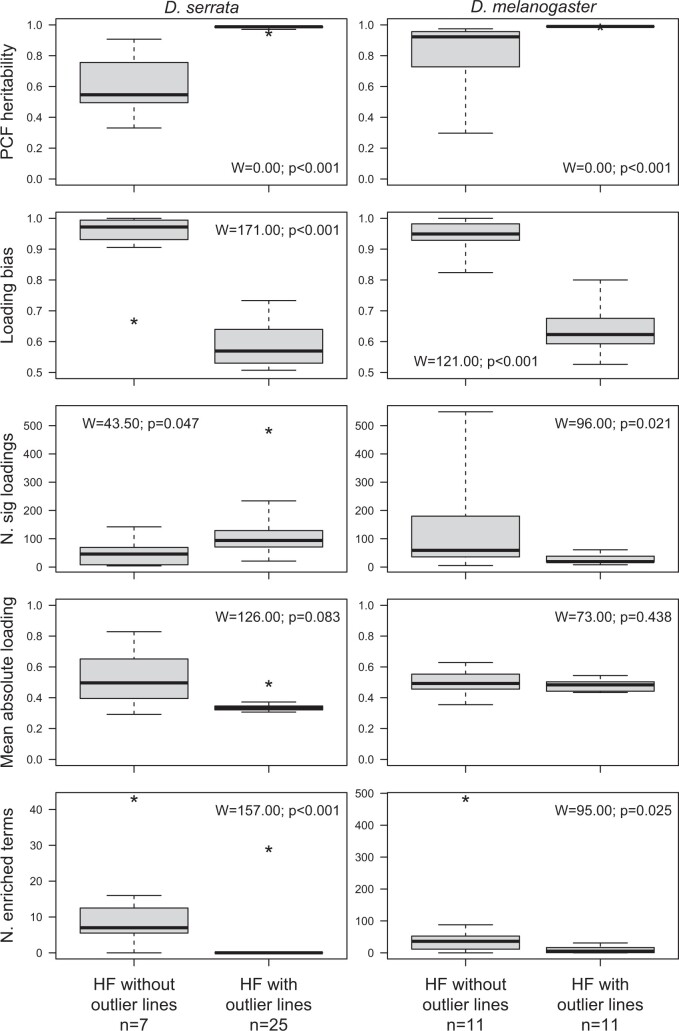
Comparison of characteristics of heritable factors with and without outliers. Bold line, box, and whiskers represent the median, 1.5 IQR, and 3 IQR, respectively. Values exceeding 3 IQR are indicated with an asterisk. We compared each characteristic between the 2 types of heritable factor (outliers absent or present) using the Wilcoxon Rank-Sum Test (results shown within each panel).

The presence or absence of outlier lines was associated with several differences in the nature of the heritable factor. In both species, heritable factors with outliers exhibited higher heritability (Fig. 6, top row) and less bias in the direction of significant loadings (Fig. 6, second row). Specifically, for heritable factors with no outliers, most significantly loading traits were influenced in the same direction, corresponding to an axis of variation contrasting lines that upregulated this set of genes with lines that downregulated expression of all these genes, relative to the population mean expression levels (e.g. Fig. 4, top right; Supplementary Figs. 4 and 5). In contrast, heritable factors with outliers exhibited a more symmetrical distribution of loadings (e.g. Fig. 4, middle and bottom right; Supplementary Figs. 4 and 5).

The number of traits significantly influenced by a heritable factor also significantly varied with the presence or absence of outlier lines, but not consistently between the species. In *D. serrata*, heritable factors with outliers had more significant loadings (Fig. 6, left panel, 3rd row), while in *D. melanogaster* they had fewer (Fig. 6, right panel, 3rd row). The average magnitude of significant trait loadings did not differ significantly between heritable factors with vs without outliers for either species (Fig. 6, 4th row). Thus, where a trait was significantly influenced by a heritable factor, the proportion of phenotypic variance in the trait attributable to that heritable factor (approximated as the square of the trait loading) was, on average, not significantly different for heritable factors with vs without outliers.

**Fig. 7.**
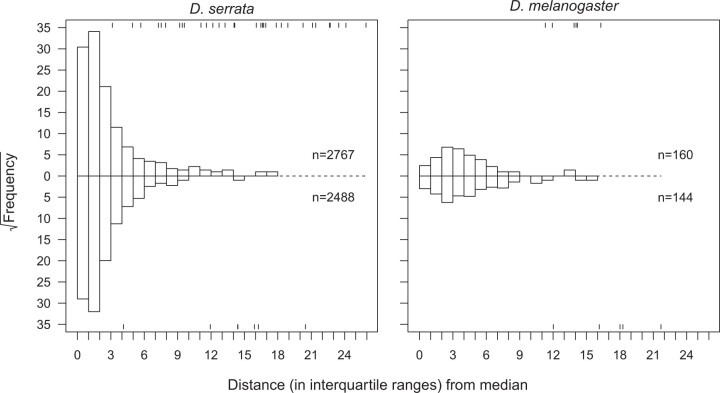
Directionality of outlier heritable latent trait values and of the associated gene expression traits. Latent trait line means (on the IQR scale) for outlier lines are shown as whiskers at the top and bottom of each panel, corresponding to positive and negative deviations from the median, respectively. For the subset of observations associated with this set of latent factors and outlier lines, the reflected histograms show the (square root) frequency of IQR-scaled individual expression trait line means that deviate above or below the median (at *y* = 0). The total counts of positive and negative deviations are shown above and below the median, respectively, in each panel.

Having observed that heritable factors with outliers had significantly less bias in the direction of their individual trait loadings (Fig. 6, 2nd row), we further investigated the distribution of the extreme values on these factors, and the individual expression traits they were predicted to affect. Note that the imposed trait loading directionality (see *Methods*) allowed us to infer positive (negative) extreme latent trait values to indicate an overall increase (decrease) in variance-standardized gene expression when summed across the traits significantly associated with the factor. In the 25 heritable factors with outliers in *D. serrata*, 33 of the 40 outlier values deviated above the median (Fig. 7a;Supplementary Fig. 1). No directional bias was apparent across the 11 heritable factors with outliers in *D. melanogaster*, although the most extreme values deviated below the median (Fig. 7b, Supplementary Fig. 2). In both species, there were slightly more (52%) individual trait values deviating above than below the median (Fig. 7), where for *D. serrata* more of the extreme deviations were above the median (consistent with the pattern observed for the heritable factors themselves: Fig. 7a), while in *D. melanogaster* the most extreme values were biased toward decreased expression (again consistent with the pattern for the heritable factors: Fig. 7b).

### Is coexpression on heritable factors consistent with physical linkage?

In *D. serrata*, we investigated whether genes coassociated with the same heritable factor colocalized in the genome. Observed chromosome frequencies did deviate significantly from expected frequencies for 7 of the 32 heritable factors (Supplementary Table 6). However, in only one case were all genes associated with a given heritable factor colocalized to the same chromosome (arm): 10 traits associated with heritable factor 31 did not map to a major chromosome, suggesting they occur on Y or 4 (Supplementary Table 6). Notably, heritable factors for which gene colocalization was statistically supported represent both those with extreme outlier lines (heritable factors 2, 4, 20, 22, and 23) and those without outliers (heritable factors 26 and 31; Supplementary Table 6).

In *D. melanogaster*, we investigated whether colocalization of genes within an inversion could account for the large changes in gene expression in the lines with outlying latent trait values. However, there was no consistent association between outlier status and inversion karyotype for any of the heritable factors with outliers (Supplementary Table 7). For any given inversion and factor combination, there were either no outlier lines with the inversion, or there was at least one representative of each class of line (with vs without an outlier) that had one or more copies of the inversion.

### Functional characteristics of heritable factors

In *D. serrata*, 6 of 7 heritable factors without outliers were significantly enriched for terms in one or more of the GO categories (BP—biological process, CC—cellular component, and MF—molecular function; Supplementary Table 8). Heritable factor 29 was associated with the largest number of terms, which broadly related to development. Of the 25 heritable factors with outliers, only heritable factor 22 was significantly enriched, with 29 BP terms relating to meiosis, and the detection of stimulus or taxis. In *D. melanogaster*, of the 11 heritable factors without outliers, 10 were significantly enriched for terms in at least one of the GO categories (Supplementary Table 8). Eight of the 11 heritable factors with outliers were significantly enriched for terms from one or more of the BP or MF categories, while terms from the CC category were notably absent; the overall number of enriched terms was substantially lower for heritable factors with outliers than those without (Supplementary Table 8; Fig. 6, bottom row). Given the larger number of trait loadings for *D. serrata* heritable factors with vs without outliers (Fig. 6), we considered whether this could have caused the pattern of differential enrichment observed. However, we note the numbers of genes in the focal and background lists are key parameters of the hypergeometric test, accounting for differences in gene number among focal lists. Furthermore, despite the overall mean difference in gene number, heritable factors across both classes had comparable numbers of genes in the analyses. For example, heritable factors 27–29 (without outliers) were associated with the largest number of enriched terms for *D. serrata* and had gene sets ranging in size from 44 to 123 genes (Supplementary Table 8). Heritable factors 4–16, 18, and 19 (with outliers) all had gene set sizes within this range but were not enriched for any terms in the GO categories (Supplementary Table 8).

## Discussion

In this study, we interrogated the multivariate distribution of genetic variance in quantitative phenotypes to characterize patterns of shared variation among traits. Here, we consider the implications of these multivariate observations for the maintenance of genetic variance in natural populations, focusing on theoretical considerations of the multivariate distribution of effect sizes. For individual traits, a rare direct test of HoC over Gaussian mutation models (Hodgins-Davis *et al.* 2015), along with observations from genetic mapping studies in humans and model taxa (Battle *et al.* 2014; Bloom *et al.* 2019), suggest rare mutations with large fitness effects contribute strongly to the maintenance of genetic variance. Consistent with this emerging body of evidence, we also detected lines (genotypes) with extreme values for individual gene expression traits in our analyses of datasets from the 2 species of *Drosophila*. Importantly, we also observed rare, extreme genotypes when examining multivariate combinations of these individual traits.

At least half of the heritable factors observed in the 2 independent datasets (species) had at least one extreme genotype (line), resulting in higher heritability of these multivariate traits relative to other multivariate traits lacking extreme genotypic values. While extreme latent trait values typically occurred for lines (genotypes) with a higher proportion of individual trait outliers, individual traits could be extreme without resulting in extreme latent trait values for the heritable factor that they were influenced by, and conversely, extreme latent trait values were observed when the heritable factor did not cause extreme values of any individual trait (Fig. 5). Thus, the presence of rare, extreme values for multivariate expression traits could not be simply predicted from inspection of the individual trait distributions.

Given several simplifying assumptions, we use the frequency of these large effect variants to make some tentative observations concerning the strength of selection in the natural *Drosophila* populations. First, we presume that extreme trait values are caused by single loci, where for heritable factors these loci have pleiotropic effects. *Trans*-regulatory factors can influence expression of large numbers of genes (Brem *et al.* 2002; Albert *et al.* 2018; Cesar *et al.* 2018), and generate extensive genetic covariation of expression (Denver *et al.* 2005; Lukowski *et al.* 2017). Therefore, it is plausible that the observed patterns of covariation reflect trans-acting pleiotropic alleles. Chromosomal location of loci, or inheritance of chromosomal inversions, were not consistent with loci affected by the same heritable factor being in close physical linkage, but nonetheless, we cannot exclude chance sampling of extreme alleles at independent loci (and the resulting transient linkage) as causing the covariance captured by the heritable factors; we further consider the nature of the heritable factors below.

Given the presumption of a single (pleiotropic) locus, and the observation that, for most individual and latent traits, only one extreme line was observed, we infer that alleles with (homozygous) major effects were segregating in the base population at a frequency of *q *≈* *1% (∼1 in the ∼120 genomes sampled to found the panel of 30 lines in these diploid species). Applying equation (2.13) of Falconer and Mackay (1996), s=μq2, where *μ* is the genic mutation rate, predicts that the strength of selection required to maintain *q* at 1% was ∼0.01–0.1, given *μ* of 10^−5^–10^−6^ (Lynch and Walsh 1998). If the true *q* is in fact lower (higher) than our sample suggests, *s* must be stronger (weaker) to maintain the allele frequency, assuming μ remains the same. Genomic mutation rates are heterogeneous across the genome (Nachman and Crowell 2000; Hodgkinson and Eyre-Walker 2011; Smith *et al.* 2018; Nesta *et al.* 2021), and mutations with larger effects might occur more rarely than smaller effect mutations (Mackay *et al.* 1992; Davies *et al.* 1999; Eyre-Walker and Keightley 2007; Heilbron *et al.* 2014; McGuigan, Collet, McGraw *et al.* 2014; Kim *et al.* 2017); such variability, resulting in higher (lower) genic mutation rates would correspond to stronger (weaker) selection to maintain *q* ∼ 1%. Finally, this prediction assumes that the large-effect alleles are recessive; if the observed outlier lines in fact carry alleles with additive or dominant effects, then the resultant increased visibility to selection means that selection 50–100 times weaker could maintain alleles at 1% frequency (Falconer and Mackay 1996, equations 2.14 and 2.15).

The predicted strength of selection (*s* ∼ 0.01–0.1) detailed above is within the range estimated for new mutations affecting fitness traits, such as viability and fecundity, based on the ratio of mutational to genetic variance (∼0.02: Houle *et al.* 1996), and consistent with the estimated average deleterious effect of heterozygous lethal genes in wild and laboratory *Drosophila* populations (∼0.02: Crow and Temin 1964). The ratio of mutational to genetic variance was also used to estimate *s* for smaller sets (5) of gene expression traits in the *D. serrata* lines analyzed here, inferring selection within the same range for 5D multivariate axes of expression (median *s *=* *0.032), but weaker selection on the individual gene expression traits (median *s *=* *0.005; McGuigan, Collet, Allen, *et al.* 2014). Nonetheless, while the strength of selection inferred to be required to maintain *q* ∼ 1% is consistent with other evidence, we need further information on mutation rates specific to loci that have been characterized as large effect variants, and on the frequency spectrum of those alleles in natural populations, to gain further insight into the mutation–selection interaction underpinning extreme multivariate trait observations.

Puzzlingly, we observed that while enrichment analyses identified coexpression patterns associated with gene functions for heritable factors without outliers, there was little evidence of common function among heritable factors with outliers, particularly in *D. serrata*. The 2 classes of heritable factor also differed in the pattern of coexpression. Heritable factors lacking outliers showed biased direction of influence on individual expression traits, corresponding to lines with relatively high vs low expression of all affected traits. This is consistent with a previous analysis of these *D. serrata* lines, where Blows *et al.* (2015) applied a matrix-building approach to estimate a single multivariate axis of genetic variation in expression of 8,750 expression traits. This coexpression axis was characterized by biased direction of trait loadings (indicating co-ordinated up-down regulation of many genes), and was enriched for multiple GO terms related to transcriptional regulation (Blows *et al.* 2015).

Heritable factors with outliers were less likely to be enriched for specific functions and exhibited patterns among lines where expression was elevated for some traits but depressed for others (i.e. unbiased direction of loadings). There are several possible nonexclusive explanations for these observations, which our data cannot further distinguish among. Heritable factors with outliers might capture gene coexpression caused by a mixture of so-called horizontal (e.g. shared chromatin status) and vertical (e.g. shared transcription factors) processes. Van Dyke *et al.* (2021) recently demonstrated that genomic “hotspots,” containing QTL with *trans*-effects on multiple expression traits (eQTL) cause coordinated changes in expression of functionally unrelated genes via horizontal mechanisms. Second, the observed pattern (large phenotypic effect, apparently unrelated function) of heritable factors with outliers could reflect extreme values of pleiotropic effects. The distribution of effects of pleiotropic alleles across multiple traits is largely unknown, but might be expected to include alleles with very weak effects on some trait(s) (Hill and Zhang 2012; Paaby and Rockman 2013); the heritable factor outlier patterns observed in this study could reflect rare pleiotropic alleles, with effects in the extreme tails of the distribution of joint effects. Alleles with such extreme pleiotropic effects might be selectively eliminated, or subject to epistatic modification to limit the range of biological processes influenced. Thus, a third plausible explanation of these data is that epistatic effects on gene expression might generate large phenotypic outliers through sampling effects (chance segregation within a single line of an unusual combination of alleles across physically unlinked loci), or, as suggested by Mackay (2014), the presence of recent mutation (i.e. rare allele) for which the population has yet to evolve epistatic amelioration of effects.

Quantitative genetic theories of the maintenance of genetic variance that incorporate indirect (apparent) stabilizing selection assume that mutations have directionally biased effects on fitness itself (decreasing it), but that the pleiotropic effects of those mutations on other traits are unbiased, equally frequently increasing as decreasing trait values (Barton 1990; Kondrashov and Turelli 1992; Johnson and Barton 2005). Empirical data to assess the assumption are sparse. For the relatively well-studied trait of size, studies in several taxa suggest that mutations typically decrease body size (Keightley and Ohnishi 1998; Lynch *et al.* 1998; Azevedo *et al.* 2002; Estes *et al.* 2005; Ostrow *et al.* 2007), with larger effect (Santiago *et al.* 1992) or more deleterious (McGuigan and Blows 2013) mutations being particularly biased. Intensive study of mutational effects in *Saccharomyces cerevisiae* found that mutations more frequently increased than decreased expression of 2 of the 10 genes studied, while a third gene exhibited the opposite bias, with more mutations decreasing expression (Hodgins-Davis *et al.* 2019). Thus, directional bias of mutational effects might be more prevalent than appreciated. Here, in *D. serrata*, for heritable factors with outliers (and to a lesser extent for the individual traits influenced by these factors), expression was biased upward; the question of emergent strong bias in the multivariate distribution of phenotypic effects warrants further investigation.

### Conclusions

Resolution of theory predicting the maintenance of genetic variation for quantitative phenotypes depends on better insight into the distributions of (pleiotropic) allelic effects on multiple traits and fitness. Widespread availability of data on many expression traits from the same genotypes provides a particularly powerful system for investigating shared variation among high-dimensional phenotypes, while the intermediary causal nature of expression traits, connecting genotype to more complex traits (Li *et al.* 2017), adds to their appeal. Our results suggest that the growing evidence that genetic variance might be due predominately to relatively large effect variants, consistent with HoC mutation models, might extend to multivariate gene expression phenotypes. However, it also remains to be determined whether the simple genetic basis of the large latent factors inferred here could be peculiar to gene expression traits, where the potential for hierarchical control of gene regulation would lend itself to master regulators of expression. Therefore, further investigations of other types of traits are required to determine whether complex trait covariances are typically consistent with large effect mutation contributing strongly to standing genetic covariance.

## Supplementary Material

iyac122_Supplemental_Tables

iyac122_Supplemental_Figures

## Data Availability

The full *Drosophila serrata* dataset is available at the Gene Expression Omnibus (GEO) database under accession number GSE45801. We used the *Drosophila melanogaster* data as summarized for the original demonstration of the BSFG presented by Runcie and Mukherjee (2013). The subsets of 3,385 expression traits measured in 2 replicate pools of male RNA from each of 30 lines analyzed here, for both species, along with the R code to implement the BSFG analyses and generate the parameters considered in this manuscript, can be downloaded from UQ eSpace (https://doi.org/10.48610/a3c5652). Supplemental material is available at *GENETICS* online.
