# Supplementary material for: Maintenance of quantitative genetic variance in complex, multitrait phenotypes: the contribution of rare, large effect variants in 2 Drosophila species
Source: Genetics. 2022 Aug 12;222(2):iyac122. doi: 10.1093/genetics/iyac122 (PMC9526065; doi:10.1093/genetics/iyac122)
Supplement: iyac122_Supplemental_Tables [file iyac122_supplemental_tables.pdf]

Table S1. Bayesian Sparse Factor parameters and their prior distributions.

Model parameter	Prior distribution	Hyperparameter values
**Λ** – *p* x *k* matrix of loadings of the *k* factors on the *p* traits; λ_{ij} is the loading of factor *j* on trait *i*.	λ_{ij} ~N$(0, \phi_{ij}^{-1}\tau_i^{-1})$, where:<br>$\phi_{ij}$~Ga$(^{\upsilon}/_{2}, ^{\upsilon}/_{2})$<br>$\tau_i = \prod_{l=1}^{i} \delta_l$<br>$\delta_1$~Ga$(a_1, b_1)$<br>$\delta_l$~Ga$(a_2, b_2)$,<br>($l$ = 2…k).	$\upsilon = 2$<br>a_1 = 2.1<br>b_1 = 1/20<br>a_2 = 3<br>b_2 = 2
$\mathbf{\Sigma}_{\mathbf{\Lambda}h^2}$ – *k* x *k* diagonal matrix of factor heritabilities; $h^2_{\mathbf{\Lambda}j}$ is the heritability of the *j*th factor.	prob($h^2_{\mathbf{\Lambda}j}$=0) = 0.5<br>prob($h^2_{\mathbf{\Lambda}j}$=$\frac{f}{n_h}$) = $\frac{1}{2(n_h-1)}$,<br>(j = 1…k, f = 1…(n_h - 1)).	n_h = 100
**Ψ** – *p* x *p* diagonal matrix of specific variances; ψ_i is the component of variance in trait *i* not explained by the factors.	ψ_i ~ $\Gamma^{-1}(v\text{-}1, Vv)$	*v=3, V=1/6*
$\mathbf{\Sigma}_{\mathbf{\Psi}h^2}$ – *p* x *p* diagonal matrix of heritabilities associated with the specific variances; $h^2_{\Sigma i}$ is the heritability associated with the specific component of trait *i*.	prob($h^2_{\Sigma i} = \frac{f}{n_h}$) = $\frac{1}{n_h}$,<br>(j = 1…p, f = 0…$(n_h - 1)$).	$n_h = 100$

Table S2. Count of expression traits with outlying lines for the two *Drosophila* gene expression datasets. Overall, 132 (3.9%) gene expression traits in *D. serrata* and 228 (6.7%) in *D. melanogaster* had at least one outlier line (defined in Table 1). Most traits had only a single outlier line, but this ranged up to four (for two traits in *D. serrata*) or six (one trait in *D. melanogaster*). We further assessed the presence of outlier lines for traits associated with one or both of two different classes of heritable factor (HF; Figures 4, 6, S1, S2) where the latent traits themselves were either associated with outlier lines (HFs with outlier lines) or not (HFs without outlier lines). For example, out of the 132 traits with outliers in *D. serrata*, 127 are associated with at least one HF; 119 of these are associated only with HFs with outliers, none are associated only with an HF without outliers, and eight are associated with at least one of each type of HF.

Species	Trait subset	Number of trait outliers								Total outlier observations
		0	≥ 1	1	2	3	4	5	6	
D. serrata	All traits	3253	132	95	23	12	2	-	-	185
	HFs either type	1894	127	90	23	12	2	-	-	180
	HFs with outlier lines	1561	119	84	21	12	2	-	-	170
	HFs without outlier lines	182	-	-	-	-	-	-	-	0
	HFs both types	151	8	6	2	-	-	-	-	10
D. melanogaster	All traits	3157	228	177	38	7	3	2	1	302
	HFs either type	1343	153	112	29	7	3	1	1	214
	HFs with outlier lines	102	94	65	19	6	3	1	-	138
	HFs without outlier lines	1182	31	24	5	1	-	-	1	43
	HFs both types	59	28	23	5	-	-	-	-	33

Table S3. Frequency of strong genetic correlations in *D. serrata* and *D. melanogaster*. We compared the relative frequency of trait pair correlations between the observed and randomized data for each species (Figure 2). In *D. serrata* (*D. melanogaster*), large correlations (i.e., $|r| > 0.5$) were approximately 10 (20) times more frequent in the observed data than the randomized data (see Ratio column). This pattern was particularly pronounced for the most extreme positive correlations, with the largest relative difference occurring at $r > 0.9$ for both species.

D. serrata				*D. melanogaster*			
Interval	Observed	Randomised	Ratio	Interval	Observed	Randomised	Ratio
[-1, -0.9]	0.0000873%	0.0000086%	10.2	[-1, -0.9]	0.0004016%	0.0000086%	46.9
(-0.9, -0.8]	0.0037713%	0.0001182%	31.9	(-0.9, -0.8]	0.0481369%	0.0001704%	282.5
(-0.8, -0.7]	0.0532526%	0.0013296%	40.1	(-0.8, -0.7]	0.3649811%	0.0018363%	198.8
(-0.7, -0.6]	0.2780484%	0.0241311%	11.5	(-0.7, -0.6]	0.9217763%	0.0254958%	36.2
(-0.6, -0.5]	0.8426656%	0.2256753%	3.7	(-0.6, -0.5]	1.7160257%	0.2279733%	7.5
(0.5, 0.6]	2.0981873%	0.2261779%	9.3	(0.5, 0.6]	3.7021032%	0.2281834%	16.2
(0.6, 0.7]	0.9974823%	0.0245395%	40.6	(0.6, 0.7]	2.0624295%	0.0257418%	80.1
(0.7, 0.8]	0.4536074%	0.0014595%	310.8	(0.7, 0.8]	1.0280371%	0.0019103%	538.2
(0.8, 0.9]	0.2065328%	0.0001788%	1155.2	(0.8, 0.9]	0.4430442%	0.0001783%	2485.3
(0.9, 1]	0.0500924%	0.0000173%	2898.0	(0.9, 1]	0.0531478%	0.0000084%	6341.7
All |r|>0.5	4.9837274%	0.5036357%	9.9	All |r|>0.5	10.3400833%	0.5115064%	20.2

Table S4. Number of traits associated with estimated factors in the *D. serrata* gene expression dataset. For each factor, we report the total number of significant trait loadings, then partition these associated traits by how many factors these traits are associated with (i.e., if the trait associates uniquely with that factor, then they appear in the count for "1", if they also associate with one another factor they contribute to the count under "2" and so on). Factors are ordered by the magnitude of their predicted contribution to phenotypic variation. We further classify factors into three types (second column): factors that were not significantly heritable (NHF), heritable factors (HF) and heritable factors with trait profiles indistinguishable from that expected from sampling error (HF*). HFs (highlighted in bold) are numbered because they were subject to further analyses. For each of the HF, we also partition the associated traits by how many HF they contributed to. The bottom row summarizes these numbers across all factors and heritable factors.

			Number of factors a trait loaded onto								Number of HFs a trait loaded onto					
Factor	Type	Total	1	2	3	4	5	6	7	8	1	2	3	4	5	6
1	NHF	771	95	224	257	144	41	10	0	0	-	-	-	-	-	-
2	NHF	622	98	216	171	95	32	9	1	0	-	-	-	-	-	-
3	NHF	249	41	103	71	24	8	1	1	0	-	-	-	-	-	-
**4**	**HF 1**	**483**	**30**	**120**	**177**	**111**	**35**	**8**	**2**	**0**	**197**	**192**	**73**	**18**	**2**	**1**
5	NHF	762	64	219	248	152	60	15	3	1	-	-	-	-	-	-
6	NHF	223	18	77	80	35	8	5	0	0	-	-	-	-	-	-
7	NHF	259	32	81	81	46	15	3	1	0	-	-	-	-	-	-
8	NHF	253	18	72	78	62	20	3	0	0	-	-	-	-	-	-
9	NHF	264	21	77	95	50	16	4	0	1	-	-	-	-	-	-
**10**	**HF 2**	**234**	**14**	**60**	**82**	**51**	**21**	**4**	**1**	**1**	**94**	**93**	**34**	**12**	**1**	**0**
11	NHF	176	22	64	53	25	10	2	0	0	-	-	-	-	-	-
12	NHF	121	16	43	40	19	1	2	0	0	-	-	-	-	-	-
**13**	**HF 3**	**193**	**14**	**47**	**74**	**42**	**8**	**7**	**0**	**1**	**73**	**81**	**26**	**11**	**1**	**1**
14	NHF	326	22	99	109	66	22	7	1	0	-	-	-	-	-	-
**15**	**HF 4**	**136**	**6**	**40**	**51**	**29**	**8**	**2**	**0**	**0**	**54**	**54**	**20**	**8**	**0**	**0**
**16**	**HF 5**	**104**	**3**	**26**	**34**	**28**	**6**	**5**	**2**	**0**	**32**	**47**	**18**	**6**	**1**	**0**
**17**	**HF 6**	**157**	**4**	**34**	**62**	**32**	**19**	**4**	**2**	**0**	**54**	**62**	**25**	**14**	**1**	**1**

**18**	**HF 7**	**136**	**7**	**38**	**45**	**31**	**10**	**4**	**0**	**1**	**45**	**65**	**17**	**7**	**2**	**0**
19	NHF	76	10	24	32	8	1	1	0	0	-	-	-	-	-	-
**20**	**HF 26**	**46**	**10**	**26**	**8**	**2**	**0**	**0**	**0**	**0**	**37**	**7**	**1**	**1**	**0**	**0**
**21**	**HF 27**	**86**	**18**	**29**	**26**	**7**	**5**	**1**	**0**	**0**	**56**	**21**	**8**	**1**	**0**	**0**
**22**	**HF 8**	**129**	**7**	**31**	**44**	**29**	**15**	**3**	**0**	**0**	**46**	**53**	**21**	**7**	**2**	**0**
**23**	**HF 9**	**113**	**8**	**24**	**46**	**23**	**9**	**3**	**0**	**0**	**41**	**44**	**21**	**4**	**2**	**1**
**24**	**HF 10**	**120**	**6**	**26**	**50**	**26**	**11**	**0**	**1**	**0**	**48**	**44**	**22**	**5**	**1**	**0**
25	NHF	165	8	52	58	33	8	4	2	0	-	-	-	-	-	-
**26**	**HF 11**	**107**	**3**	**26**	**33**	**28**	**12**	**4**	**1**	**0**	**28**	**43**	**25**	**9**	**2**	**0**
**27**	**HF 12**	**100**	**6**	**24**	**34**	**21**	**10**	**4**	**0**	**1**	**31**	**43**	**18**	**5**	**2**	**1**
**28**	**HF 28**	**142**	**12**	**27**	**50**	**39**	**11**	**3**	**0**	**0**	**47**	**58**	**27**	**10**	**0**	**0**
**29**	**HF 13**	**83**	**4**	**19**	**28**	**16**	**13**	**3**	**0**	**0**	**27**	**32**	**16**	**6**	**1**	**1**
**30**	**HF 14**	**94**	**5**	**22**	**29**	**25**	**10**	**3**	**0**	**0**	**34**	**35**	**15**	**9**	**1**	**0**
**31**	**HF 29**	**53**	**14**	**16**	**16**	**6**	**1**	**0**	**0**	**0**	**23**	**24**	**6**	**0**	**0**	**0**
**32**	**HF 15**	**85**	**5**	**25**	**33**	**15**	**5**	**2**	**0**	**0**	**33**	**31**	**15**	**4**	**1**	**1**
33	NHF	56	11	14	19	6	5	1	0	0	-	-	-	-	-	-
**34**	**HF 16**	**83**	**8**	**17**	**32**	**18**	**7**	**1**	**0**	**0**	**28**	**30**	**19**	**5**	**0**	**1**
**35**	**HF 17**	**53**	**1**	**13**	**18**	**14**	**7**	**0**	**0**	**0**	**16**	**22**	**13**	**2**	**0**	**0**
**36**	**HF 18**	**78**	**4**	**16**	**25**	**18**	**11**	**3**	**1**	**0**	**28**	**27**	**15**	**5**	**2**	**1**
**37**	**HF 19**	**79**	**1**	**21**	**25**	**22**	**8**	**2**	**0**	**0**	**25**	**30**	**17**	**5**	**2**	**0**
**38**	**HF 20**	**71**	**9**	**14**	**20**	**18**	**7**	**3**	**0**	**0**	**29**	**19**	**15**	**6**	**0**	**2**
39	NHF	76	9	22	26	19	0	0	0	0	-	-	-	-	-	-
40	NHF	141	9	38	49	31	9	5	0	0	-	-	-	-	-	-
**41**	**HF 21**	**44**	**2**	**12**	**10**	**14**	**2**	**3**	**1**	**0**	**16**	**13**	**9**	**5**	**1**	**0**
**42**	**HF 22**	**28**	**0**	**11**	**11**	**1**	**2**	**3**	**0**	**0**	**10**	**11**	**3**	**2**	**1**	**1**
43	NHF	45	2	18	14	5	3	3	0	0	-	-	-	-	-	-
**44**	**HF 23**	**30**	**3**	**7**	**13**	**3**	**3**	**1**	**0**	**0**	**12**	**9**	**8**	**0**	**1**	**0**
45	NHF	70	4	20	23	12	7	3	0	1	-	-	-	-	-	-

**46**	**HF 30**	**6**	**1**	**1**	**1**	**3**	**0**	**0**	**0**	**0**	**3**	**3**	**0**	**0**	**0**	**0**
47	NHF	16	2	8	4	2	0	0	0	0	-	-	-	-	-	-
48	NHF	31	1	15	9	6	0	0	0	0	-	-	-	-	-	-
**49**	**HF 24**	**22**	**2**	**5**	**6**	**6**	**2**	**1**	**0**	**0**	**9**	**7**	**4**	**1**	**1**	**0**
**50**	**HF 25**	**21**	**1**	**3**	**13**	**2**	**0**	**1**	**0**	**1**	**7**	**10**	**2**	**0**	**2**	**0**
51	HF*	6	3	3	0	0	0	0	0	0	-	-	-	-	-	-
**52**	**HF 31**	**10**	**2**	**5**	**3**	**0**	**0**	**0**	**0**	**0**	**7**	**3**	**0**	**0**	**0**	**0**
53	NHF	11	1	3	3	2	1	0	1	0	-	-	-	-	-	-
54	NHF	35	0	16	17	2	0	0	0	0	-	-	-	-	-	-
**55**	**HF 32**	**4**	**3**	**0**	**1**	**0**	**0**	**0**	**0**	**0**	**3**	**1**	**0**	**0**	**0**	**0**
56	HF*	2	2	0	0	0	0	0	0	0	-	-	-	-	-	-
57	NHF	13	0	4	6	3	0	0	0	0	-	-	-	-	-	-
58	NHF	5	3	1	0	1	0	0	0	0	-	-	-	-	-	-
59	HF*	2	2	0	0	0	0	0	0	0	-	-	-	-	-	-
All factors/HFs		3274	727	1149	881	382	105	26	3	1	1193	607	171	42	6	2

Table S5. Number of traits associated with estimated factors in the *D. melanogaster* gene expression dataset. For each factor, we report the total number of significant trait loadings, then partition these associated traits by how many factors these traits are associated with (i.e., if the trait associates uniquely with that factor, then they appear in the count for "1", if they also associate with one another factor they contribute to the count under "2" and so on). Factors are ordered by the magnitude of their predicted contribution to phenotypic variation. We further classify factors into three types (second column): factors that were not significantly heritable (NHF), heritable factors (HF) and heritable factors with trait profiles indistinguishable from that expected from sampling error (HF*). HFs (highlighted in bold) are numbered because they were subject to further analyses. For each of the HF, we also partition the associated traits by how many HF they contributed to. The bottom row summarizes these numbers across all factors and heritable factors.

			Number of factors a trait loaded onto					Number of HFs a trait loaded onto		
Factor	Type	Total	1	2	3	4	5	1	2	3
**1**	**HF 12**	**549**	**282**	**208**	**50**	**9**	**0**	**454**	**90**	**5**
2	NHF	410	215	141	48	6	0	-	-	-
**3**	**HF 13**	**125**	**63**	**53**	**8**	**1**	**0**	**104**	**20**	**1**
4	NHF	275	125	107	35	7	1	-	-	-
**5**	**HF 14**	**235**	**97**	**107**	**25**	**6**	**0**	**168**	**63**	**4**
6	NHF	438	155	189	83	11	0	-	-	-
**7**	**HF 15**	**236**	**99**	**89**	**41**	**7**	**0**	**167**	**61**	**8**
**8**	**HF 16**	**65**	**32**	**24**	**8**	**1**	**0**	**50**	**14**	**1**
9	HF*	72	21	35	15	1	0	-	-	-
**10**	**HF 17**	**40**	**23**	**12**	**3**	**2**	**0**	**29**	**9**	**2**
**11**	**HF 18**	**28**	**8**	**12**	**8**	**0**	**0**	**19**	**9**	**0**
**12**	**HF 1**	**61**	**22**	**30**	**6**	**3**	**0**	**44**	**15**	**2**
**13**	**HF 19**	**59**	**32**	**19**	**7**	**1**	**0**	**44**	**15**	**0**
**14**	**HF 20**	**49**	**32**	**11**	**5**	**0**	**1**	**41**	**8**	**0**
**15**	**HF 2**	**35**	**18**	**12**	**5**	**0**	**0**	**27**	**7**	**1**
**16**	**HF 3**	**52**	**13**	**28**	**10**	**0**	**1**	**32**	**18**	**2**
17	HF*	45	15	20	7	3	0	-	-	-

18	HF*	37	13	12	12	0	0	-	-	-
**19**	**HF 4**	**42**	**13**	**14**	**12**	**3**	**0**	**21**	**18**	**3**
20	HF*	40	15	17	8	0	0	-	-	-
21	HF*	22	9	9	4	0	0	-	-	-
22	HF*	36	11	18	6	1	0	-	-	-
23	HF*	34	7	16	8	2	1	-	-	-
24	HF*	33	10	15	7	1	0	-	-	-
25	HF 5	23	5	10	8	0	0	13	8	2
26	HF*	25	8	8	7	1	1	-	-	-
27	HF*	27	10	11	6	0	0	-	-	-
28	HF*	32	8	19	4	1	0	-	-	-
**29**	**HF 6**	**18**	**2**	**11**	**5**	**0**	**0**	**8**	**10**	**0**
30	HF*	24	5	10	8	1	0	-	-	-
**31**	**HF 7**	**19**	**4**	**12**	**2**	**1**	**0**	**10**	**9**	**0**
32	HF*	21	5	13	3	0	0	-	-	-
33	NHF	77	22	35	18	2	0	-	-	-
**34**	**HF 8**	**18**	**5**	**6**	**7**	**0**	**0**	**9**	**9**	**0**
**35**	**HF 9**	**19**	**2**	**14**	**2**	**1**	**0**	**8**	**9**	**2**
**36**	**HF 21**	**32**	**10**	**14**	**6**	**2**	**0**	**18**	**14**	**0**
37	HF*	15	4	8	3	0	0	-	-	-
38	HF*	23	10	11	2	0	0	-	-	-
39	HF*	15	2	10	3	0	0	-	-	-
40	HF*	12	6	2	3	1	0	-	-	-
41	NHF	44	10	23	7	4	0	-	-	-
42	HF*	8	4	3	1	0	0	-	-	-
43	HF*	8	4	3	1	0	0	-	-	-
**44**	**HF 10**	**9**	**4**	**3**	**2**	**0**	**0**	**5**	**4**	**0**
**45**	**HF 11**	**8**	**1**	**4**	**3**	**0**	**0**	**1**	**7**	**0**

46	HF*	2	0	2	0	0	0	-	-	-
**47**	**HF 22**	**5**	**2**	**2**	**0**	**1**	**0**	**4**	**1**	**0**
All factors/HFs		2374	1463	716	174	20	1	1276	209	11

Table S6. Distribution across chromosomes of ESTs associated with heritable factors in the *D. serrata* lines. We mapped the 3385 analyzed ESTs to chromosomes of the reference *D. serrata* genome using BLASTn, resulting in "null" frequencies on X (446 ESTs), 2L (545), 2R (683), 3L (688), 3R (817) and "other" (206 ESTs that didn't align to any of the five largest chromosomes). We then conducted chi-square tests of the chromosome frequencies of the subsets of traits associated with each individual heritable factor (HF). HFs with observed frequencies differing significantly from the null (s < .01) are shown in bold.

	Chr X		Chr 2L		Chr 2R		Chr 3L		Chr 3R		Other		χ^2	p	s
HF	Obs	Exp	Obs	Exp	Obs	Exp	Obs	Exp	Obs	Exp	Obs	Exp			
1	51	63.64	71	77.77	117	97.46	95	98.17	126	116.58	23	29.39	9.3	0.099	0.037
**2**	**39**	**30.83**	**30**	**37.68**	**51**	**47.21**	**35**	**47.56**	**73**	**56.48**	**6**	**14.24**	**17**	**0.005**	**0.001**
3	23	25.43	25	31.07	44	38.94	41	39.23	54	46.58	6	11.75	6.1	0.292	0.093
**4**	**10**	**17.92**	**23**	**21.9**	**44**	**27.44**	**33**	**27.64**	**23**	**32.82**	**3**	**8.28**	**20.9**	**0.001**	**<0.001**
5	9	13.7	15	16.74	14	20.98	30	21.14	33	25.1	3	6.33	12.1	0.034	0.012
6	10	20.69	30	25.28	33	31.68	35	31.91	40	37.89	9	9.55	6.9	0.228	0.069
7	13	17.92	29	21.9	20	27.44	36	27.64	31	32.82	7	8.28	8.5	0.131	0.046
8	10	17	30	20.77	23	26.03	32	26.22	28	31.14	6	7.85	9.4	0.095	0.032
9	8	14.89	22	18.19	24	22.8	26	22.97	26	27.27	7	6.88	4.5	0.479	0.127
10	5	15.81	19	19.32	23	24.21	33	24.39	34	28.96	6	7.3	11.6	0.041	0.016
11	12	14.1	16	17.23	25	21.59	24	21.75	26	25.83	4	6.51	2.1	0.829	0.217
12	15	13.18	13	16.1	23	20.18	24	20.32	22	24.14	3	6.09	3.7	0.599	0.157
13	5	10.94	11	13.36	25	16.75	20	16.87	19	20.03	3	5.05	9.2	0.102	0.041
14	14	12.39	15	15.13	22	18.97	21	19.11	18	22.69	4	5.72	2.4	0.796	0.197
15	6	11.2	10	13.69	23	17.15	23	17.28	19	20.52	4	5.17	7.7	0.175	0.061
16	5	10.94	17	13.36	23	16.75	13	16.87	22	20.03	3	5.05	8.5	0.133	0.051
17	5	6.98	8	8.53	3	10.69	13	10.77	20	12.79	4	3.23	10.8	0.055	0.022
18	3	10.28	15	12.56	22	15.74	17	15.85	20	18.83	1	4.75	11.2	0.047	0.019
19	9	10.41	9	12.72	9	15.94	21	16.06	28	19.07	3	4.81	10.7	0.058	0.027
**20**	**4**	**9.35**	**13**	**11.43**	**10**	**14.33**	**17**	**14.43**	**26**	**17.14**	**1**	**4.32**	**12.2**	**0.032**	**0.009**

21	5	5.8	6	7.08	4	8.88	13	8.94	13	10.62	3	2.68	5.4	0.373	0.114
**22**	**0**	**3.69**	**3**	**4.51**	**2**	**5.65**	**17**	**5.69**	**2**	**6.76**	**4**	**1.7**	**35.5**	**<0.001**	**<0.001**
**23**	**3**	**3.95**	**5**	**4.83**	**13**	**6.05**	**6**	**6.1**	**3**	**7.24**	**0**	**1.83**	**12.5**	**0.028**	**0.006**
24	2	2.9	6	3.54	3	4.44	5	4.47	5	5.31	1	1.34	2.6	0.759	0.177
25	0	2.77	2	3.38	5	4.24	4	4.27	9	5.07	1	1.28	6.6	0.253	0.084
**26**	**0**	**6.06**	**3**	**7.41**	**4**	**9.28**	**1**	**9.35**	**3**	**11.1**	**35**	**2.8**	**395.4**	**<0.001**	**<0.001**
27	12	11.33	18	13.85	19	17.35	12	17.48	22	20.76	3	5.23	4.2	0.523	0.141
28	21	18.71	26	22.86	24	28.65	31	28.86	37	34.27	3	8.64	5.5	0.355	0.103
29	4	6.98	11	8.53	10	10.69	18	10.77	10	12.79	0	3.23	10.7	0.057	0.025
30	1	0.79	1	0.97	1	1.21	1	1.22	1	1.45	1	0.37	1.4	0.927	0.239
**31**	**0**	**1.32**	**0**	**1.61**	**0**	**2.02**	**0**	**2.03**	**0**	**2.41**	**10**	**0.61**	**154.3**	**<0.001**	**<0.001**
32	0	0.53	1	0.64	0	0.81	0	0.81	3	0.97	0	0.24	6.9	0.23	0.077

Table S7. Karyotypes for five inversions and HF score outlier status for 29 of the 30 *D. melanogaster* lines. Five known inversions were segregating as one or two copies across 11 of the 29 karyotyped lines that were included in the BSFG analysis. All outlier lines for HFs 1, 2 and 6-8 had the standard karyotype (ST/ST) for each of the five inversions. The outlier line for each of HFs 3, 4, 5 and 9-11 carried at least one copy (kayrotypes ST/INV or INV/INV) of one (HFs 4,5,9-11) or two (HF 3) types of inversion. In each of these cases, at least one line falling within the bulk of the distribution for that HF also carried at least one copy of the inversion (shown in bold).

		Inversion Karyotype										
HF	Line	In.2L.t		In.2R.NS		In.3R.P		In.3R.K		In.3R.Mo		
	Type	ST/ST	INV/INV	ST/ST	INV/INV	ST/ST	INV/INV	ST/ST	ST/INV	ST/ST	ST/INV	INV/INV
1	Bulk	26	2	26	2	27	1	27	1	22	1	5
	Outlier	1	0	1	0	1	0	1	0	1	0	0
2	Bulk	26	2	26	2	27	1	27	1	22	1	5
	Outlier	1	0	1	0	1	0	1	0	1	0	0
3	Bulk	27	**1**	26	2	27	1	27	1	23	1	**4**
	Outlier	0	**1**	1	0	1	0	1	0	0	0	**1**
4	Bulk	26	2	27	**1**	27	1	27	1	22	1	5
	Outlier	1	0	0	**1**	1	0	1	0	1	0	0
5	Bulk	26	2	26	2	27	1	27	1	23	1	**4**
	Outlier	1	0	1	0	1	0	1	0	0	0	**1**
6	Bulk	26	2	26	2	27	1	27	1	22	1	5
	Outlier	1	0	1	0	1	0	1	0	1	0	0
7	Bulk	26	2	26	2	27	1	27	1	22	1	5
	Outlier	1	0	1	0	1	0	1	0	1	0	0
8	Bulk	26	2	26	2	27	1	27	1	22	1	5
	Outlier	1	0	1	0	1	0	1	0	1	0	0
9	Bulk	26	2	26	2	27	1	27	1	23	1	**4**
	Outlier	1	0	1	0	1	0	1	0	0	0	**1**
10	Bulk	27	**1**	26	2	27	1	27	1	22	1	5
	Outlier	0	**1**	1	0	1	0	1	0	1	0	0
11	Bulk	26	2	26	2	27	1	27	1	23	**0**	**5**

Outlier	1	0	1	0	1	0	1	0	0	**1**	**0**

Table S8. Summary of functional enrichment analyses conducted using g:Profiler and semantic similarity analysis conducted with GOSemSim. Only HFs with significant enrichment for at least one term in any of the three Gene Ontology (GO) categories are shown. The full lists of significantly enriched terms can be downloaded at https://doi.org/10.48610/90441fc.

			Number of enriched terms			
Species	HF Type	HF#	GO:BP	GO:CC	GO:MF	Theme(s)
D. serrata	HF with outliers	22	29	-	-	Development
	HF without outliers	26	-	7	-	Regulation
		27	-	6	-	Membrane
		28	4	9	3	Membrane, transport
		29	21	12	10	Meiosis, detection of stimulus, taxis
		30	-	-	9	Transmembrane transport
		32	3	-	2	Metabolic process
D. melanogaster	HF with outliers	2	2	-	2	Metabolic process
		4	-	-	1	Dynein complex binding
		6	30	-	-	Immune response
		7	-	-	5	Enzyme activity
		8	13	-	12	Development of photoreceptor cells

	9	4	-	27	Transmembrane transporter activity
	10	-	-	8	Binding
	11	-	-	8	Telomeric DNA binding
HF without outliers	12	3	11	-	Mitochondrial/organelle membrane
	13	-	1	-	Egg chorion
	14	4	44	2	Mitochondrial ribosome
	15	449	25	9	Regulation of biological processes, cellular communication, dendrite development and synapse assembly, actin cytoskeleton organisation, endocytosis and localisation of molecules, cell migration and motility, morphogenesis.
	17	51	37	-	Endocytosis and localisation of molecules, regulation of transport, cell communication, synaptic signalling.
	18	6	3	-	Metabolic process
	19	31	20	4	ATP metabolic process, muscle system process, cuticle development, muscle cell development, organic acid metabolism, cellular respiration.

	20	29	-	11	Cellular carbohydrate metabolism, phenol-containing compound metabolism, response to fungus, peptide secretion, amino acid metabolism
	21	30	3	3	Photoreceptor cell maintenance, sensory perception of smell, receptor metabolic process, organic acid metabolic process, cellular response to abiotic stimulus, adaptation of signalling pathway, cellular respiration.
	22	13	1	-	Immune response
