# Supplementary material for: Maintenance of quantitative genetic variance in complex, multitrait phenotypes: the contribution of rare, large effect variants in 2 Drosophila species
Source: Genetics. 2022 Aug 12;222(2):iyac122. doi: 10.1093/genetics/iyac122 (PMC9526065; doi:10.1093/genetics/iyac122)
Supplement: iyac122_Supplemental_Figures [file iyac122_supplemental_figures.pdf]

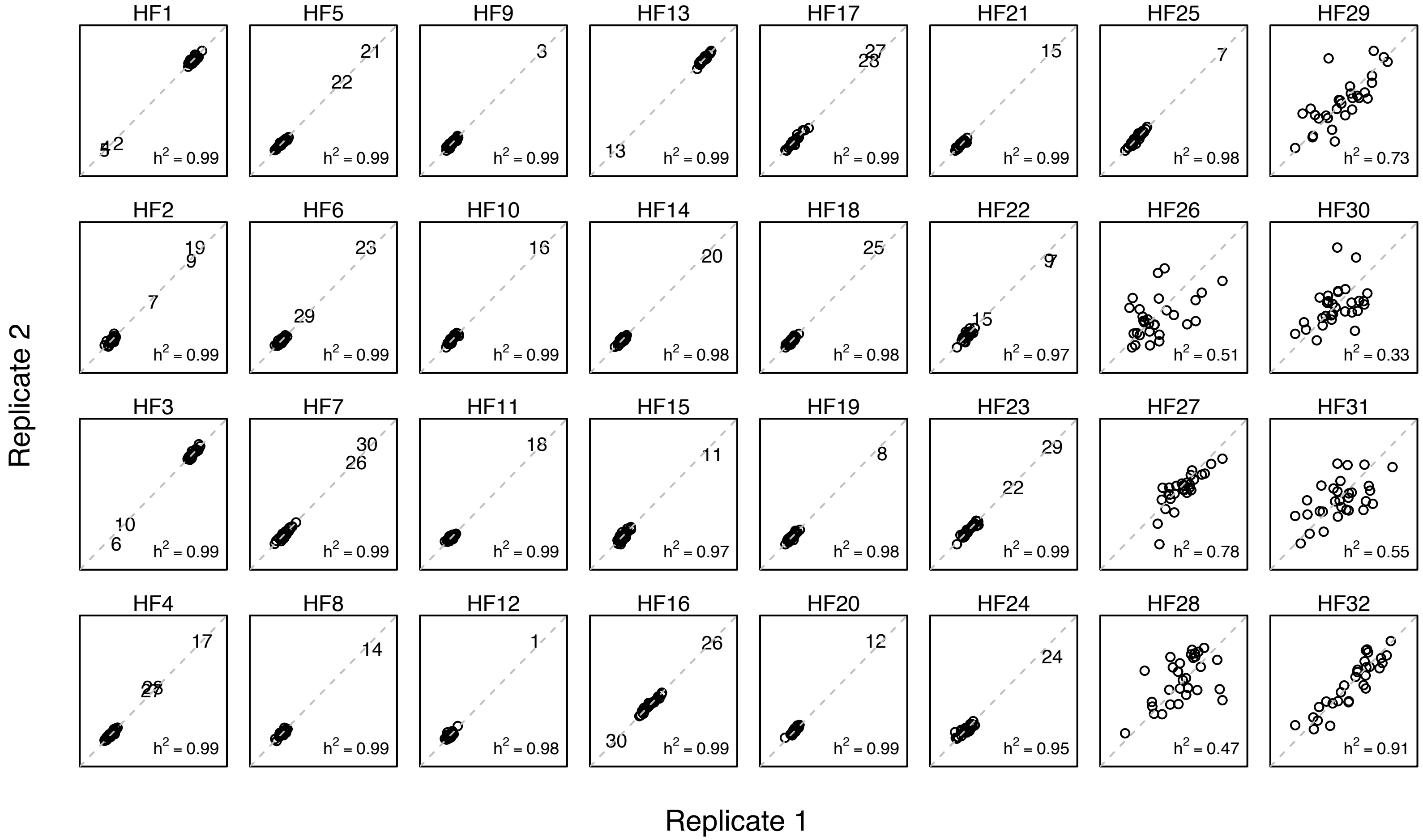

Figure S1. Latent trait values for replicate pairs of the 30 *D. serrata* lines for the 32 heritable factors. Replicate-level latent trait values are presented. Each panel is labeled above with the factor ID, as shown in Table S4. Heritable factors (HF) are ordered first by type (HF1-HF25 have outlier line(s), while HF26-HF32 do not) then by decreasing contribution to phenotypic variance. Numbered values are identifiers for individual lines that met the criteria for being outliers (see Methods; Table 1) on that factor; notably, no individual line was an outlier for more than two heritable factors (i.e., the same line identifier is not consistently repeated across panels). The dashed line indicates a 1:1 relationship between

replicate latent trait values, where tighter clustering of latent trait values along this line is associated with higher heritability (heritability shown in the bottom right of each panel), and low replicate-specific (non-genetic) influences. Several HFs have two lines with almost identical latent trait values: lines 4 and 5 for HF1; lines 27 and 28 for HF4 (and to a lesser extent, HF 17); and lines 7 and 9 for HF22.

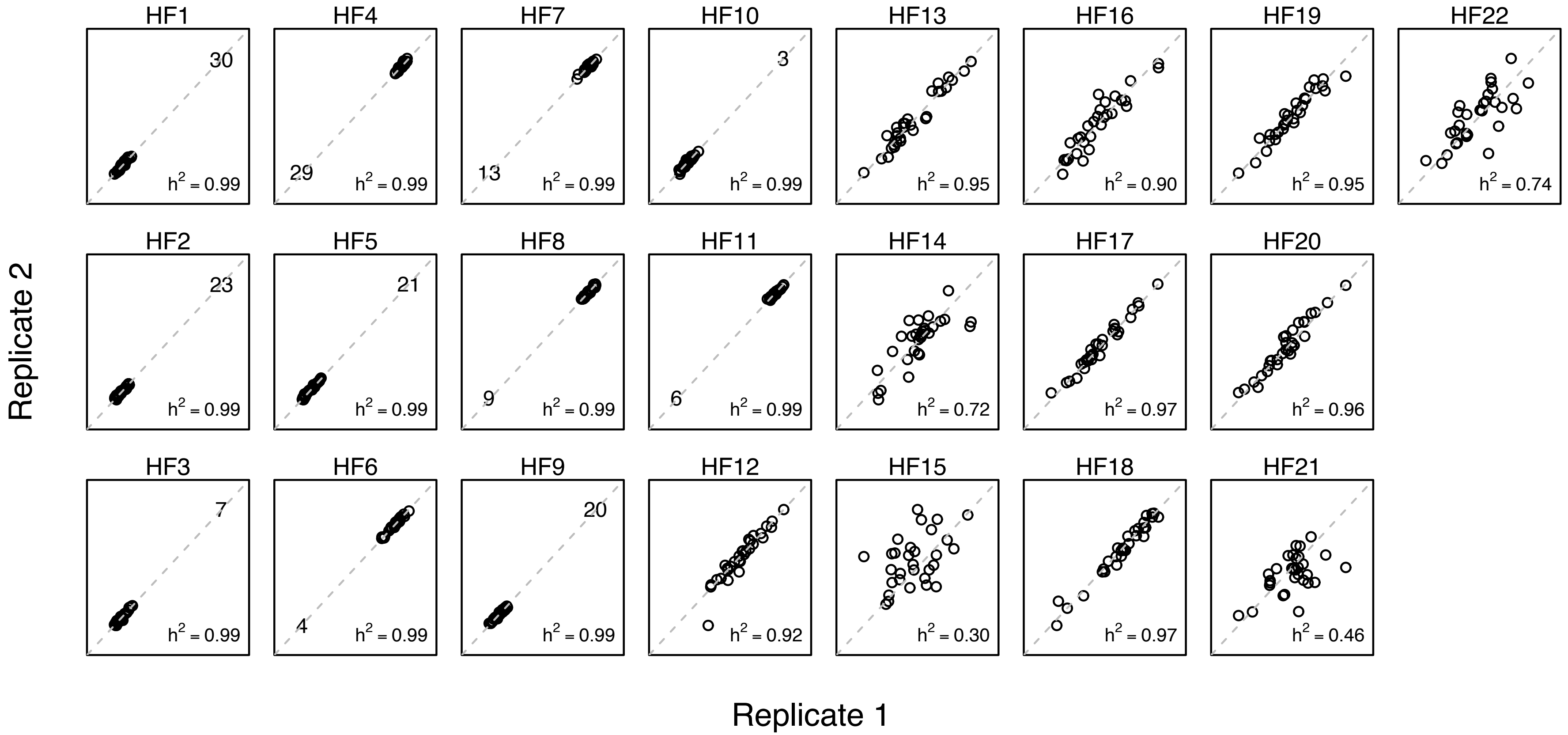

Figure S2. Latent trait values for replicate pairs of the 30 *D. melanogaster* lines for the 22 heritable factors. Replicate-level latent trait values are presented. Each panel is labeled above with the factor ID, as shown in Table S5. Heritable factors (HF) are ordered first by type (HF1-HF11 have outlier line(s), while HF11-HF22 do not) then by decreasing contribution to phenotypic variance. Numbered values are identifiers for individual inbred lines that met the criteria for being outliers (see Methods; Table 1) on that factor; notably, the same individual line was not an outlier across many HF (i.e., the same line identifier is not consistently repeated across panels). The dashed line indicates a 1:1 relationship between replicate latent trait values, where tighter clustering of latent trait values along this line is associated with higher heritability (heritability shown in the bottom right of each panel), and low replicate-specific (non-genetic) influences.

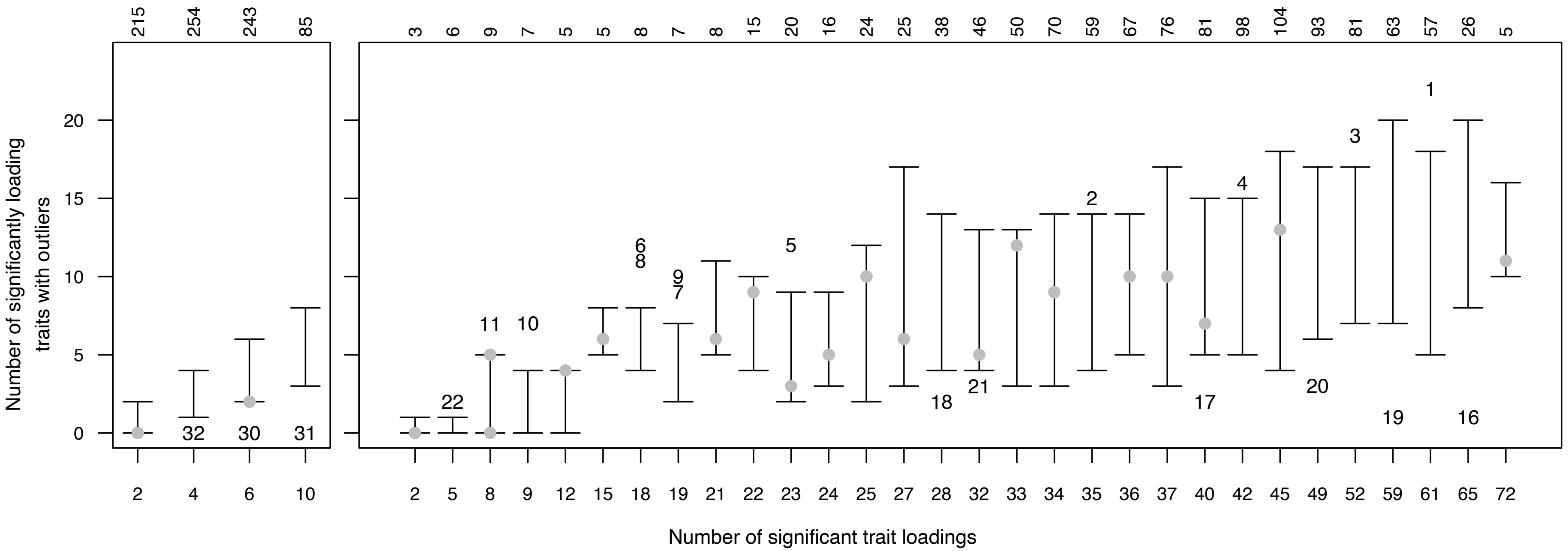

Figure S3. Influence of individual trait outlier lines on estimation of heritable factors in randomized datasets, and comparison to observed data for *D. serrata* (A) and *D. melanogaster* (B)**.** For a given observed heritable factor (Tables S4, S5), we first identified the subset of heritable factors in the randomized data with the same number of significant trait loadings (bottom x-axis); the size of the comparison subset, across all factors in the 100 randomized datasets, is shown on the top axis. We then compared the number of significantly loading traits with outlying lines (y-axis) between the observed factor and the matched subset of randomized data factors. We excluded from subsequent investigation any observed factors (plotted as grey shaded points) that fell within the range typically observed in the randomized data factors. Where the number of traits with outlier lines for an observed heritable factor fell outside the 95% range of the corresponding randomized data subset (labelled, in black, as per heritable factor numbers in Tables S4 and S5), we retained the observed factor for further investigation. We thereby excluded three *D. serrata* factors (note, two factors with two significant trait loadings each are superimposed) and 20 *D. melanogaster* factors (note, two factors with 15 significant trait loadings each are superimposed) from subsequent analyses.

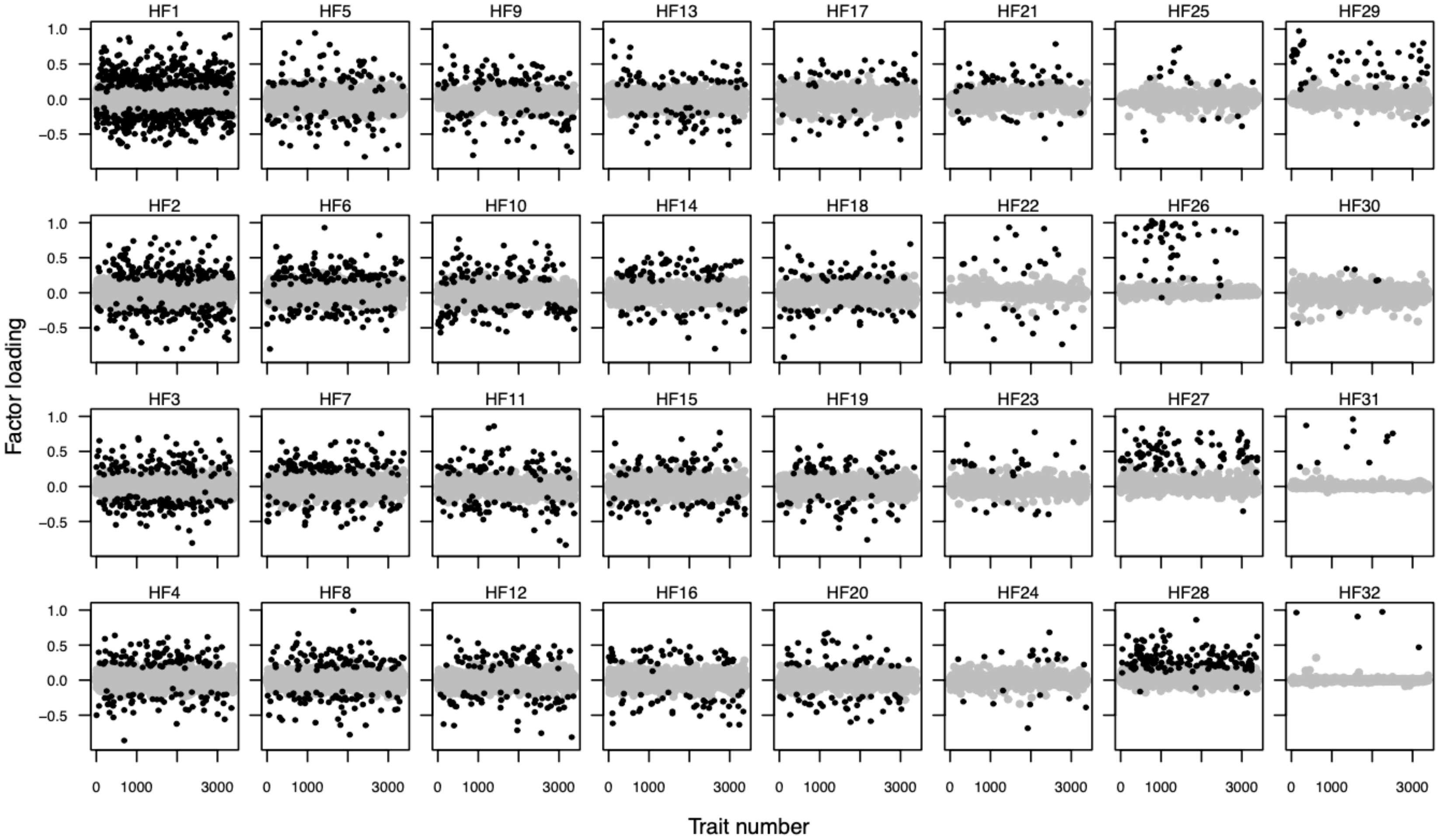

Figure S4. Trait loadings of the 32 heritable *D. serrata* latent factors. Significant (nonsignificant) trait loadings are represented by black (grey) circles for each heritable factor (HF). Traits are plotted in order of EST number, where traits were arbitrarily assigned a numerical identifier prior to analyses. Factors exhibiting the greatest degree of directional bias in trait loadings (HFs 26-32) are also those without outlier lines (Fig. S1).

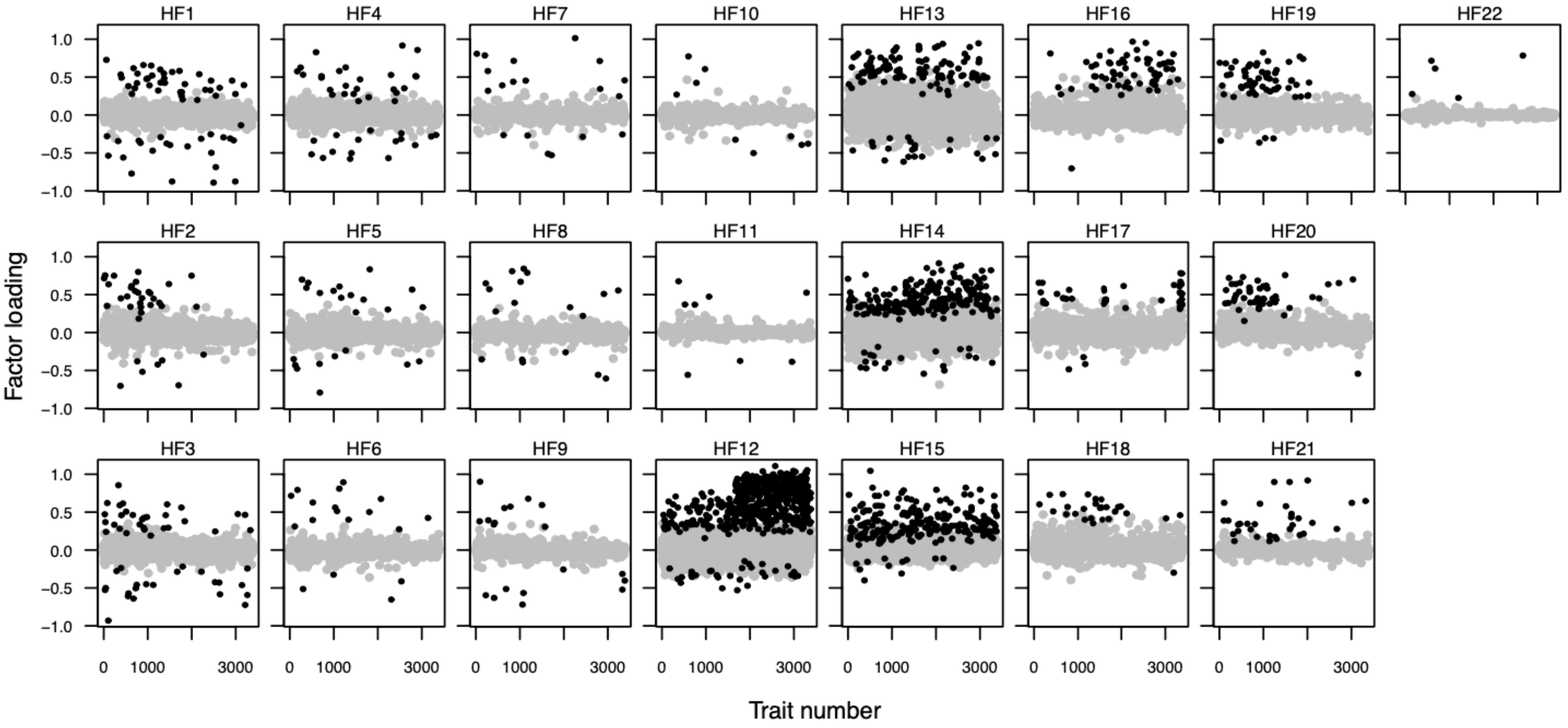

Figure S5. Trait loadings of the 22 heritable *D. melanogaster* factors. Significant (nonsignificant) trait loadings are depicted as black (grey) circles. Traits are plotted as ordered in the full dataset, which was arranged by module membership (D. Runcie, pers. comm). Notably, this ordering of traits reveals apparent relationships between the heritable factors (HF) identified here, and co-associating sets of expression traits previously identified by Ayroles et al. (2009). Specifically, HFs 12 and 16 appear to relate strongly to one of the two large modules detected in the original gene expression analysis (i.e., higher numbered traits contribute), while HFs 2, 19 and 20 appear to be strongly associated with the other (i.e., lower numbered traits contribute). As we observed for *D. serrata*, HFs with the largest degree of directional bias (HFs 12-22) are also those without outlier lines (Fig. S2).
